# Normal human adipose tissue functions and differentiation in patients with biallelic *LPIN1* inactivating mutations

**DOI:** 10.1194/jlr.P075440

**Published:** 2017-10-06

**Authors:** Michele Pelosi, Eric Testet, Soazig Le Lay, Isabelle Dugail, Xiaoyun Tang, Guillaume Mabilleau, Yamina Hamel, Marine Madrange, Thomas Blanc, Thierry Odent, Todd P. W. McMullen, Marco Alfò, David N. Brindley, Pascale de Lonlay

**Affiliations:** Centre de Référence des Maladies Héréditaires du Métabolisme,* Institut Imagine des Maladies Génétiques, Laboratoire de génétique des maladies autoinflammatoires monogéniques, INSERM UMR1163, Université Paris Descartes et Hôpital Necker-Enfants malades (Assistance publique – Hôpitaux de Paris), Paris, France; Laboratoire de Biogenèse Membranaire-UMR 5200,† CNRS, Université de Bordeaux, Villenave d’Ornon, France; INSERM,§ UMR1063, Université d’Angers, UBL, Angers, France; INSERM,** U1166, Equipe 6, Université Pierre et Marie Curie, Paris, France; Department of Biochemistry,†† Signal Transduction Research Group, University of Alberta, Edmonton, Alberta, Canada; SCIAM,§§ Université d’Angers, Angers, France; Department of Pediatric Surgery and Urology,*** Hôpital Necker-Enfants malades-Université Paris Descartes, Sorbonne Paris Cité, Paris, France; Department of Pediatric Orthopedics,††† Hôpital Necker-Enfants malades-Université Paris Descartes, Sorbonne Paris Cité, Paris, France; Department of Surgery,§§§ University of Alberta, Edmonton, Alberta, Canada; Dipartimento di Scienze Statistiche,**** Sapienza Università di Roma, Rome, Italy

**Keywords:** adipocytes, gene expression, lipodystrophies, phosphatases/lipid, inborn errors of metabolism, human lipids, adipogenic differentiation, human fat lipid analysis

## Abstract

Lipin-1 is a Mg^2+^-dependent phosphatidic acid phosphatase (PAP) that in mice is necessary for normal glycerolipid biosynthesis, controlling adipocyte metabolism, and adipogenic differentiation. Mice carrying inactivating mutations in the *Lpin1* gene display the characteristic features of human familial lipodystrophy. Very little is known about the roles of lipin-1 in human adipocyte physiology. Apparently, fat distribution and weight is normal in humans carrying *LPIN1* inactivating mutations, but a detailed analysis of adipose tissue appearance and functions in these patients has not been available so far. In this study, we performed a systematic histopathological, biochemical, and gene expression analysis of adipose tissue biopsies from human patients harboring *LPIN1* biallelic inactivating mutations and affected by recurrent episodes of severe rhabdomyolysis. We also explored the adipogenic differentiation potential of human mesenchymal cell populations derived from lipin-1 defective patients. White adipose tissue from human *LPIN1* mutant patients displayed a dramatic decrease in lipin-1 protein levels and PAP activity, with a concomitant moderate reduction of adipocyte size. Nevertheless, the adipose tissue develops without obvious histological signs of lipodystrophy and with normal qualitative composition of storage lipids. The increased expression of key adipogenic determinants such as *SREBP1*, *PPARG*, and *PGC1A* shows that specific compensatory phenomena can be activated in vivo in human adipocytes with deficiency of functional lipin-1.

Studies in vitro and in vivo using mouse models show that the protein lipin-1, encoded by the gene *Lpin1*, is an enzyme necessary for normal glycerolipid biosynthesis, which is also able to control adipocyte metabolism and adipogenic differentiation (1–7). Lipin proteins act as Mg^2+^-dependent phosphatidate phosphatases (PAPs) that catalyze the dephosphorylation of phosphatidate (PA) to diacylglycerol, a direct precursor of triacylglycerols (TAGs) and polar lipids (PLs), such as phosphatidylethanolamine and phosphatidylcholine (8–10). Although lipin-1 does not have a DNA-binding motif, it is able to translocate to the nucleus of hepatocytes and adipocytes, where it can act as a transcriptional coregulator (3, 11–14), interacting with PGC1α/PPARα and PPARγ, modulating their transcriptional activity and regulating adipogenesis, FA oxidation (FAO), and the expression of key components of the mitochondrial respiratory chain (11–13, 15). Accordingly, lipin-1-deficient mice exhibit reduced expression of hepatic PPAR-regulated genes and impaired FAO activity (5, 13, 16). The effects of lipin-1 on transcription apparently do not require lipin-1 PAP activity, demonstrating that the enzymatic activity of lipin-1 can be functionally separated from its ability to coregulate the transcription (11–13, 17). Moreover, lipin-1 has also been implicated in the modulation of pro-inflammatory responses, both repressing nuclear factor of activated T-cells cytoplasmic 4 activity and downstream cytokine production in adipocytes (15) and positively controlling the production of pro-inflammatory factors during macrophage activation (18, 19).

*Lpin1* fatty liver dystrophic (fld) mice, i.e., *Lpin1^(fld/fld)^*-deficient mice, carrying spontaneous inactivating mutations in the *Lpin1* gene, show the characteristic features of the human congenital lipodystrophy (1–3, 7). This condition consists of a group of genetic disorders characterized by severe loss of body fat associated with metabolic disturbances such as fatty liver, hypertriglyceridemia, and insulin resistance (20). The absence or decreased levels of PAP activity in white adipose tissue (WAT) of *Lpin1^(fld/fld)^* mice as well in the WAT of other mouse or rat models without a functional lipin-1 result in dysregulated TAG biosynthesis, with subsequent accumulation of PA (2, 5, 7, 14, 21–24). The lipodystrophic lipin-1-deficient mouse models are characterized by: *i*) impaired maintenance of mature adipocytes with severe reduction in TAG storage and consequent severe reduction (about 80%) of body fat mass; *ii*) the presence of abnormal adipocytes with multilocular lipid droplets; and *iii*) impairment in adipogenic differentiation with a substantial increase in the presence of immature adipocytes in the WAT (5, 7, 14). The rat model with a dominant negative mutation in the *Lpin1* gene (the *Lpin1^1Hubr^* rats) presents a milder but analogous form of lipodystrophy, characterized by impaired expression of key transcriptional adipogenic factors such as PGC1α and PPARα (23). Unexpectedly, the *Lpin1^(fld/fld)^* mouse does not show defects in TAG synthesis or β-oxidation in the heart where lipin-1 provides about 80% of the PAP activity and in this case, the presence of the paralogues lipin-2 or lipin-3 appears to compensate for the absence of lipin-1 (24).

The accumulated evidence from observations in lipin-1-deficient rodent models presents a lipodystrophic phenotype, together with data showing that lipin-1 is involved in the adipogenesis in vitro (3, 4, 6, 25). The observations that the gene encoding for lipin-1 is prevalently expressed in adipose tissue in human beings and mouse (9) make *LPIN1* a top-rank candidate for human lipodystrophy. However, we and other investigators have reported that the presence of *LPIN1* inactivating mutations in human patients lead to a dramatic and peculiar skeletal muscle-related pathological phenotype, which consists of recurrent and severe episodes of rhabdomyolysis (i.e., massive breakdown of the skeletal muscle fibers) without any evident involvement of the adipose tissue (26–28). Consistent with the prevailing human phenotype, the absence of lipin-1 can induce a failure in the maintenance of muscle integrity under special conditions of metabolic stress in *Lpin1^(fld/fld)^* mice (29).

The role of lipins in human adipogenesis and human adipose tissue functions is still undefined. Recently, it was shown in the human Simpson-Golabi-Behmel syndrome (SGBS) preadipocyte cell line (an in vitro cell model for studies of human adipocyte differentiation) that lipin-1 is progressively induced during adipogenesis and that depletion of lipin-1 by siRNA leads to depletion of about 95% of PAP activity and a significant reduction of the key adipogenic transcription factors PPARG and SREBP1 (30). Nevertheless, the SGBS cell line differentiated normally and accumulated neutral lipids under lipin-1 deficiency, supporting the hypothesis of the existence of alternative pathways for TAG synthesis in human adipocytes subjected to conditions of repressed lipin-1 expression (30).

In-depth biochemical, histological, and biological analyses of adipose tissue derived from lipin-1 defective patients have not been performed so far. Despite the absence of obvious signs of adipose tissue loss and lack of the metabolic disturbances usually associated with lipodystrophy, we cannot yet exclude that human lipin-1-defective individuals (as in rodents) manifest some failures of adipose tissue homeostasis. To clarify the effects of lipin-1 deficiency on human adipose tissue, we performed a systematic analysis of WAT biopsies derived from patients carrying biallelic *LPIN1* inactivating mutations. Also, to assess the possible role played by lipin-1 in human adipogenesis, we triggered the adipogenic differentiation potential of primary mesenchymal cells derived from lipin-1-defective patients.

Our analysis established that the adipose tissue from human lipin-1-defective patients is characterized by a marked decrease in lipin-1 protein levels and PAP activity with a concomitant moderate reduction of adipocyte size. However, WAT in lipin-1-defective human individuals develops without detectable histopathological signs of lipodystrophy and with an apparently normal qualitative composition of stored lipids. The increased expression of key adipogenic determinants such as *SREBP1*, *PPARG*, and *PGC1A* shows that a specific compensatory phenomenon can be activated in vivo in human adipocytes in the presence of depleted lipin-1 expression.

## MATERIALS AND METHODS

### Materials

All reagents were purchased from Sigma-Aldrich unless otherwise specified.

### Patients and healthy control individuals

All patients were clinically and genetically characterized and had experienced recurrent episodes of rhabdomyolysis (26–28, 31). When fat biopsies were taken, the patients were apparently in good health, fully metabolically compensated, and had not experienced an episode of rhabdomyolysis for several months. **Table 1** summarizes the characterization of the patients and healthy control individuals used in the present study.

**TABLE 1.** Characterization of the patients and healthy controls individuals used in the study

Individual	Specimens Used in Experiment:	Age	Sex	*LPIN1* Mutation Nucleotides	*LPIN1* Mutation Amino Acids
Control 1	s.a.t.b.	12	F	WT	WT
Control 2	s.a.t.b.	15	F	WT	WT
Control 3	s.a.t.b.	18	M	WT	WT
Control 4	s.a.t.b.	16	M	WT	WT
Control 5	s.a.t.b.	4	M	WT	WT
Control 6	s.a.t.b.	15	M	WT	WT
Control 7	s.a.t.b.	15	M	WT	WT
Control 8	Skin fibroblast population	5	F	WT	WT
Control 9	Skin fibroblast population	2.5	M	WT	WT
Control 10	Skin fibroblast population	8.5	M	WT	WT
Lipin-1-deficient-1	s.a.t.b.	5	M	c.1162C>T	p.Arg388X
				c.1162C>T	p.Arg388X
Lipin-1-deficient-2	s.a.t.b.	5	M	c.1441+2T>C	p.Asn417LysfsX22
				c.2295-866_2410-30del	p.Glu766_Ser838del
Lipin-1-deficient-3	s.a.t.b.	11	M	c.1441+2T>C	p.Asn417LysfsX22
				c.2295-866_2410-30del	p.Glu766_Ser838del
Lipin-1-deficient-4	s.a.t.b.	47	M	c.1162C>T	p.Arg388X
				c.1162C>T	p.Arg388X
Lipin-1-deficient-5	Skin fibroblast population	4	F	c.2295-863_2410-27del	p.Glu766_Ser838del
				c.2295-863_2410-27del	p.Glu766_Ser838del
Lipin-1-deficient-6	Skin fibroblast population	11.5	F	c.1441+2T>C	p.Asn417LysfsX22
				c.2295-863_2410-27del	p.Glu766_Ser838del
Lipin-1-deficient-7	Skin fibroblast population	4	F	c.2295-863_2410-27del	p.Glu766_Ser838del
				c.921delT	p.Gln308ArgfsX36

s.a.t.b., subcutaneous adipose tissue biopsy.

### Biopsy method

Subcutaneous WAT samples biopsies were obtained from the brachial region (deltoid region) of lipin-1-defective patients and from the dorsal region of control individuals, with the exception (shown in Fig. 4) of two WAT control biopsies from breast adipose tissue from healthy adult individuals (aged 53 and 56) subjected to reduction mastoplasty. Otherwise, the collection of the WAT samples was performed using a <0.5 cm scalpel incision, and a 14-gauge needle for the subsequent collection of the biopsy. Adipose tissue samples were rapidly rinsed in PBS and immediately analyzed or frozen. This work was approved by the Hôpital Necker-Enfant Malades Ethical Committee after declaration to the *Département de la Recherche Clinique et du Développement* (Assistance Publique, Hôpitaux de Paris, France) and informed consent was obtained (from a parent or guardian for each child). Breast adipose tissue (controls B1 and B2 in Fig. 4) was obtained with approval of the University of Alberta Health Research Ethics Board ID Pro00018758, with written informed consent.

### Electron microscopy

WAT biopsies (∼8 mm^3^) were fixed for 16 h at 4°C in 2.5% glutaraldehyde in 0.1 M Sorensen’s buffer, pH 7.4. Samples were rinsed in 0.2 M cacodylate buffer, pH 7.4, and subsequently postfixed with 2% osmium tetroxide/1.5% potassium ferrocyanide in 0.2 M cacodylate buffer, pH 7.4, for 45 min at room temperature. Samples were then rinsed with distilled water, dehydrated in ethanol, and embedded in EMbed 812 epoxy resin (LFG Distribution) at 60°C for 48 h. Semi-thin sections (1 µm thickness) were cut with an EM-UC7 ultramicrotome (Leica), colored with 1% methylene blue/1% Azur B and observed with an Olympus AX-60 microscope (Olympus) equipped with a QImaging QIClick digital CCD camera (QImaging, Surrey, BC, Canada). Thin sections (60 nm thickness) were cut and counterstained with uranyl acetate and observed with a Jeol JEM 1400 microscope (Jeol, Croissy sur Seine, France) operated at 120 KeV and equipped with a Gatan Orius Camera (Gatan, Pleasanton, CA).

### Immunohistofluorescence

Adipose tissue biopsies (WAT) were fixed for 12–16 h at room temperature in 4% paraformaldehyde and embedded in paraffin. Five micrometer sections were cut at 50 μm intervals and mounted on charged glass slides. Paraffin was removed with xylene, and slides were stained for the expression of perilipin, using an anti-perilipin-1/PLIN1 antibody (Progen) as described (32). Adipose tissue sections were stained with DAPI prior to mounting them using the mounting medium Fluoromount-G (SouthernBiotech). The total numbers of nuclei and adipocytes were counted in five microscopic fields for each biopsy. The adipose tissue cellular composition was then determined for each histological sample as the total number of adipocytes [in which perilipin-1 decorates an unilocular lipid droplet (LD) occupying the entire cell volume] divided by the total number of nuclei (DAPI positive).

### Adipocyte diameter measurements

Fat biopies (∼8 mm^3^) were fixed as indicated in the Electron Microscopy section (see above); semi-thin sections (500 nm-thickness) were cut with an EM-UC7 ultramicrotome (Leica), colored with 1% methylene blue/1% Azur B, and observed with an AX-60 microscope as indicated above. Adipocyte diameters (expressed in micromolars) of at least 100 mature adipocytes in each section were measured on microscopic images, using Image J software. The cumulative distribution function (CDF) of adipocyte diameters was estimated statistically by the empirical CDF (see Fig. 3). The measured adipocyte diameters for each experimental individual or group were divided into arbitrary classes with ∼16 μm intervals. The Kolmogorov-Smirnov test was used as a nonparametric statistic to test the equality of CDFs; i.e., testing the null hypothesis that the samples were drawn from the same population (described by the corresponding CDF). All statistical analyses, including graphical representations and exploratory statistics, were performed using the open source software R, release 3.2.2 “Fire safety” (33).

### Enzymatic activity for PAP

An enzymatic assay for PAP was performed as described in (24). Briefly, adipose tissue was homogenized in sucrose buffer consisting of 250 mM sucrose, 0.6 mM DTT, and protease inhibitor cocktail, followed by centrifugation at 12,000 *g* for 5 min at 4°C to collect the infranatant in which PAP activity was measured. Each sample was assayed in a total volume of 100 μl consisting of 100 mM Tris/HCl (pH 7.4) containing 0.6 mM DTT, 1.5 mM MgCl_2_, 2 mg/ml fat-free BSA, protease inhibitor cocktail, 30 nM microcystin-LR, 0.6 mM PA labeled with [^3^H]palmitate {[9,10-^3^H(N)]palmitic Acid, PerkinElmer}, 1 mM EDTA/EGTA, 0.4 mM phosphatidylcholine, and 200 μM tetrahydrolipstatin to block lipase activity. Parallel measurements were performed in the absence of Mg^2+^ and in the presence of 8 mM N-ethylmaleimide to determine the contribution from lipid phosphate phosphatase (LPP) activity. The diacylglycerol that was formed was extracted in 2 ml chloroform-methanol (19:5, v/v) containing 0.08% olive oil as carrier. Activated alumina was added to remove the unreacted PA and any liberated [^3^H]palmitate from the chloroform phase. The chloroform phase was dried and the radioactivity determined. PAP activity was calculated by subtracting the NEM-insensitive LPP activity from the total phosphatidate phosphatase activity. Each sample was assayed at three different protein concentrations to ensure a proportional response.

### Western blot analysis for lipin-1

Adipose tissue was homogenized in RIPA buffer followed by centrifugation at 12,000 *g* for 10 at min 4°C. The infranatant was collected and 4 vol of ice-cold acetone were added to precipitate protein. After centrifugation at 12,000 *g* for 10 min at 4°C, protein precipitations were washed with 1 vol of ice-cold acetone and proteins were dried at room temperature. Protein precipitations were resuspended in RIPA buffer. Sixty micrograms of protein was loaded for PAGE (24) and probed with anti-lipin1/*LPIN1* antibody (Abcam) or with an anti tubulin antibody as a loading control. Blots were analyzed by Li-cor C-DiGit Blot imaging scanner.

### Lipid analysis

WAT (about 15–20 mm^3^) were extracted in 2 ml of chloroform/methanol 2:1, (v/v) using a glass Potter-Elvehjem homogenizer with a PTFE pestle. Five hundred microliters of 1% perchloric acid was added and the extracted suspension vortexed for 30 s. After centrifugation (1,000 *g* for 10 min at room temperature), the organic phase was isolated and the aqueous phase further extracted with 2 ml chloroform-methanol 2:1 (v/v). The two organic phases were pooled in a glass test tube (test tube A) and evaporated to dryness. A fully quantitative procedure to efficiently separate the less abundant total PLs from the highly prevalent TAG from total lipid extracts from each fat biopsy was adapted from a previously described two-phase hexane/(ethanol-water) extraction method (34). Briefly, a stock biphasic solvent system was prepared by mixing equal volumes of hexane and an aqueous ethanol solution (13% water, 87% ethanol, by vol). 4.5 ml of the upper phase and 1.5 ml of the lower phase of the biphasic solvent system were add to test tube A containing the dried extracted lipids, and mixed thoroughly. The extracted lower phase from the test tube A was transferred to a second test tube, test tube B, containing 4.5 ml preequilibrated upper phase stock solvent system. 1.5 ml of the lower phase of the stock solvent system was added to the test tube A. Test tubes A and B were mixed thoroughly and after equilibration the lower phase of test tube B was isolated, and the lower phase of test tube A was transferred to test tube B. The above procedure was repeated six times. The combined upper phases (i.e., eight times 1.5 ml) represented the ethanolic phase, prevalently containing polar lipids (PLs) (Fig. 3), and the remaining upper phases from test tubes A and B represented the hexane phase 1 and the hexane phase 2, respectively, prevalently containing neutral lipids (Fig. 3). The different extracted phases were evaporated to dryness and redissolved in 4 ml (for the hexane phase 1) or 100 µl (for the hexane phase 2 and the ethanolic phase) chloroform-methanol (2:1, v/v). One hundred microliters of the hexane phases and the ethanolic phase were analyzed by one-dimensional high-performance TLC on silica gel 60 (Merck) using hexane-diethyl ether-acetic acid (90:15:2, v/v/v) as mobile phase for neutral lipid separation (35, 36) and using chloroform, methanol, water, ammonia 25% (60:34:4:2, v/v/v/v) as mobile phase for the polar lipid separation (37). The lipids were then located by spraying the TLC plates with 0.001% (w/v) primuline in 80% acetone, followed by Imaging (ChemiDoc, Bio-Rad).

The silica gel zones corresponding to TAG and PL were scraped from the TLC plates and the acyl chains analyzed by GC-flame-ionization detection. The scraped spots were added to 1 ml methanol/2.5% H_2_SO_4_ containing 5 μg heptadecanoic acid methyl ester. After maintaining the lipids in the mixture at 80°C for 1 h, 1.5 ml water was added and FA methyl esters extracted using 0.75 ml hexane. Separation of FA methyl esters was performed by GC (Hewlett–Packard 5890, series II) on a 15 m × 0.53 mm Carbowax column (Alltech Associates, Deerfield, IL) by flame-ionization detection. The retention times of FA methyl esters were determined by comparison with standards, and they were quantified using heptadecanoic acid methyl ester as a standard (38).

### Isolation of dermal fibroblasts

Human primary dermal fibroblast populations were established from biopsy specimens obtained from the brachial region (deltoid region) of lipin-1 defective patients or from the dorsal forearm region of control individuals. Tissue biopsies were dissociated enzymatically in 0.25% collagenase type I and 0.05% DNase in DMEM with 20% FBS overnight at 37°C. Primary fibroblast cultures were established in 25 cm^2^ culture flasks in DMEM supplemented with 20% FCS and 2 mM glutamine. Monolayer cultures were maintained at 37°C in 10% CO_2_ until about 70% confluence. Cells were then passaged and used for experiments.

### Adipogenic differentation of human primary dermal fibroblasts populations

Human primary dermal fibroblasts were plated in growth medium (GM) containing DMEM + 2 mM glutamine and 10% FCS (all from Life Technologies) on culture dishes. After 2 days of postconfluence (which was considered as day 0), cells were shifted in adipogenic differentiation medium (DM) containing DMEM + 2 mM glutamine, 10% FCS, 500 nM bovine insulin, 500 nM dexamethasone, 500 μM 3-isobutyl-1-methylxanthine, and 50 μM indomethacin or in control medium (DMEM + 2 mM glutamine containing 10% FCS). Every 3 days, the DM or the control medium was replaced, and at 4, 8, and 12 days, cells were collected for RNA extraction and further analysis.

### RNA isolation, reverse transcription, and real-time PCR

Human primary dermal fibroblasts cells were washed with ice-cold PBS and total RNA was extracted using TRIzol reagent (Life Technologies). Human fat biopsies (WAT, ∼5–10 mm^3^) were rinsed in cold PBS and immediately frozen in liquid nitrogen; subsequently biopsies were homogenized in ∼500 ml TRIzol Reagent using a glass Potter-Elvehjem homogenizer with a glass pestle. The homogenate was subjected to centrifugation at 12,000 *g* for 8 min at 4°C. The pellet and the top fat layer were discarded whereas the aqueous phase was subjected to standard TRIzol protocol according to manufacturer’s instructions for total RNA purification. Digestion of contaminating genomic DNA was performed with DNA-free DNase treatment (Ambion). First strand cDNA was generated using a kit with random primers (High Capacity cDNA Reverse Transcription kit, Applied Biosystems) from 1 μg of the total RNA. Newly synthesized cDNA was diluted 5-fold in DNase-free water, and 5% of this cDNA was used in each real-timePCR assay performed using a 7500 Real Time PCR System (Applied Biosystems) (39, 40). The standard curve method was used to calculate the relative mRNA levels for each transcript examined and human actin β(ACTB) expression was used as a reference to normalize the results. The sequence (5′ to 3′) of the specific primers used for the SYBR Green quantification of the mRNA (Power SYBR Green PCR Master Mix, Applied Biosystems) were: Human membrane metallo-endopeptidase (MME), forward primer: CCTCACCATCATAGCTGTGACAA; reverse primer: TGACTTGCA­AA­TACCATCATCGT. Human melanoma cell adhesion molecule (MCAM), forward primer: CCTGGACTTGGACACCATGAT; reverse primer: ACGTCAGACACAT­AGTTCACCAGTAGT. Human vascular cell adhesion molecule 1 (VCAM1), forward primer: CAAAGGCAGAGTACGCAAACAC; reverse primer: GCTGACCAAGACGGTTGTATCTC. Human integrin α 11 (ITGA11), forward primer: GCACCCCATTTCCAAACAAC; reverse primer: ACCGCCTCTCATCCATGGT. Human dipeptidyl-peptidase 4 (DPP4), forward primer: CAGTCACCAATGCAACTTCCA; reverse primer: ACAAGTAGTGATCCCCTATCAACATAGA. Human PPAR γ (PPARG), forward primer: GGGCGATCTTGACAGGAAAG; reverse primer: CCCATCATTAAGG­AATTCATGTCAT. Human FASN, forward primer: CAGACGAG­AGCAC­CTTT­GATGAC; reverse primer: CAGGTCTATGAGGCC­TATCTGGAT. Human PPAR γ coactivator 1 α (PPARGC1A), forward primer: ACCCAGAACCATGCAAATCAC; reverse primer: GCTCCATGAATTCTCAGTCTTAACAA. Human LPIN1, forward primer: TGAAAAGGGGCTCTGTGGAC; reverse primer: ACTACAGA­GCTGCTTGACGG. Human LPIN2, forward primer: GGT­CCCCTTTAGAGACCACCTATC; reverse primer: CACCTCCAG­CTCTGAATCACTCTT. Human ACTB, forward primer: CTGGCACCCAGCACAATG; reverse primer: CCGATCCACACGGAGTACTTG. Human sterol regulatory element binding transcription factor 1 (SREBF1), forward primer: GCTGTCCACAAAAGCAAA­TCTCT; reverse primer: TCAGTGTGTCCTCCACCTCAGT.

## RESULTS

### Histomorphometrical and ultrastructural analyses of human adipose tissue in patients carrying biallelic *LPIN1* mutations

Mice carrying inactivating mutations of the *Lpin1* gene are characterized by manifested lipodystrophy features, with a severe reduction of total fat mass, presence of abnormal adipocytes with multilocular LDs, and a significant increase of immature adipocytes in the adipose tissue (2, 7, 14). The role of lipins in human adipogenesis is still undefined. To investigate the consequences of the deficiency of lipin-1 in human adipogenesis, we examined fat biopsies from human patients carrying biallelic inactivating mutations in the *LPIN1* gene, to search for signs of defects in the histological structure indicative of lipodystrophy.

According to previous studies, two divergent histopathological subsets can be observed in congenital or acquired forms of human lipodystrophy (41). One form of lipodystrophy is characterized histologically by: *i*) small adipocytes embedded in hyaline connective tissue, *ii*) the presence of a myxoid stroma with numerous capillaries, and *iii*) very scarce infiltration of inflammatory cells (41). The second type of human lipodystrophy is characterized instead by a distinctive “inflammation of the fat,” with the hallmark presence of focal lymphocytes, histiocytes, and plasma cells, but showing normal-appearing adipocytes and normal vasculature (41). In all cases, lipodystrophic adipose tissue can be recognized histologically by alteration in the proportion of mature fat cells to nonfat cells (32).

**Fig. 1.**
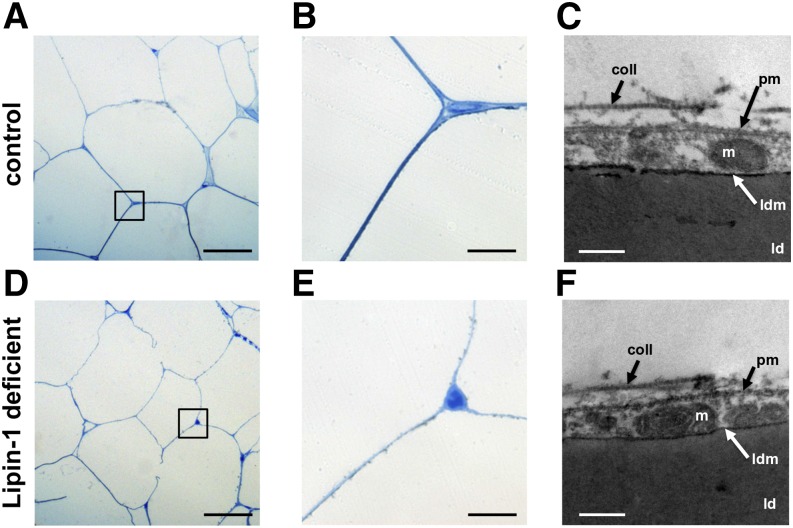
Histomorphometrical and ultrastructural analyses of the adipose tissue in patients carrying biallelic *LPIN1* inactivating mutations. A, B, D, E: microscopic imagines at low (A, D) and high (B, E) magnification of semi-thin sections stained with azur B-methylene blue, representative of subcutaneous adipose tissue biopsies derived from normal control individuals (A, B) and from lipin-1-defective patients (D, E), from biopsies fixed in glutaraldehyde and treated as indicated in the Methods section. No signs of lipodistrophy or perturbation of adipose tissue morphology and organization were observed in histological sections from lipin-1-defective patients (D, E) compared with controls (A, B). Scale bars in A and D: 25 μm; scale bars in B and E: 2.5 μm. C, F: transmission electronic microscopy microphotographs. No signs of alteration in adipocyte ultrastructure were observed in lipin-1-defective patients. Scale bars in C and F: 200 nm. m, mitochondria; coll, extracellular fibrillar collagen; ld, lipid droplet; ldm, membrane of the lipid droplet; pm, plasmamembrane. The experiment shown was repeated three times using biopsies derived from three independent lipin-1 defective patients and from three independent control individuals and gave similar results.

**Fig. 2.**
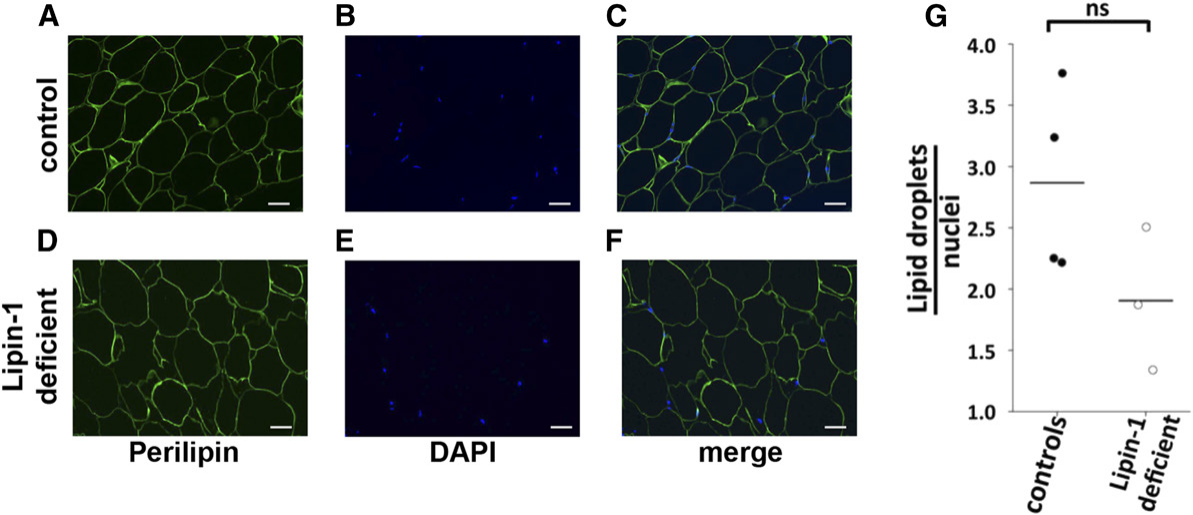
Histomorphometrical analysis of the adipose tissue in patients carrying biallelic *LPIN1* inactivating mutations. Histological sections from subcutaneous adipose tissue biopsies derived from lipin-1-defective patients and controls were immunostained with an anti-perilipin antibody; the nuclei were counterstained with DAPI. The sections were subjected to immunoflourescence microscopy. Representative immunofluorescent image for a control individual (A–C) and for a lipin-1-defective patient (D–F) are shown. Perilipin cellular distribution and relative abundance did not show significant difference in the adipose tissue from lipin-1-deficient individuals when compared with that of normal controls. Anti-perilipin antibody, green; DAPI staining of the nuclei, blue; scale bars = 25 μm. The experiment was repeated using biopsies from three independent lipin-1 defective patients and four control individuals and gave similar results. G: Ratio of the total number of adipocyes to total number of cells (estimated by nuclei staining). For each biopsy, the number of perilipin-positive adipocytes and DAPI-positive nuclei was counted in five random microscopic fields; the dots indicate the average ratio for each biopsy. Bars represent the mean from four independent control individuals and three independent lipin-1-defective patients. Student’s *t*-test and the Mann-Whitney *U* test were used for controls versus lipin-1-deficient sample comparison. ns, not significant.

Mature adipocytes (and the shape of the LDs) were identified on histological adipose tissue sections based on their characteristic perilipin-staining of the monolocular LD [(42) and Fig. 2]. Perilipin-1 was abundantly expressed in mature adipocytes, where it localized at the surface of the intracellular LDs (42). As in the normal controls, the histological and ultrastructural analyses of human fat biopsies from lipin-1-defective patients did not show any evidence of histopathological features associated with lipodystrophy (**Figs. 1**, ** 2**). No manifest signs suggestive of hypoplastic development, inflammation, increased presence of stromal cells, or perturbation of morphology (i.e., increased presence of mixoid stroma, lobules of small adipocytes, or abnormal vasculature) were observed by histological and ultrastructural analyses of adipose tissue from lipin-1-defective patients (Figs. 1, 2).

We did not observe abnormal or multilocular LDs (Fig. 2). Perilipin immunostaining of the LDs was conserved because it decorated a unilocular LD occupying the entire cell volume of the adipocytes (Fig. 2). The ultrastructural analysis confirmed the full integrity of the LD membrane and structure in lipin-1-defective patients (Fig. 1C, F).

We next assessed the ratio between the number of mature adipocytes (showing large perilipin-1-positive monolocular LD) and the number of cells (based on DAPI nuclei detection) in adipose tissue histological sections (Fig. 2). This enabled us to detect possible changes in the proportion between adipocyte and nonadipocyte cells (including the stromal cell fraction). Significant decreased values in this ratio are indicative of adipose tissue infiltration by nonadipose cells, disclosing an inflammatory state or suggesting a hypoplastic development of the adipose tissue, which are are often observed in lipodystrophies (32). The perilipin/DAPI ratio was not significantly affected in human adipose tissue from patients carrying biallelic *LPIN1* inactivating mutations compared with normal control individuals (Fig. 2). This result indicates that adipose tissue from lipin-1-defective patients was not significantly hypoplastic or infiltrated with immature adipocytes and nonadipocytic cells, including inflammatory cells.

Some forms of mild lipodystrophy are characterized by the presence of adipocytes with reduced size (41), and this feature is symptomatic of a deficiency in accumulation of TAG in LDs. We performed a semiautomated histomorphometric analysis of the diameter of the adipocytes in fat biopsies from normal controls and patients harboring *LPIN1* inactivating mutations.

**Fig. 3.**
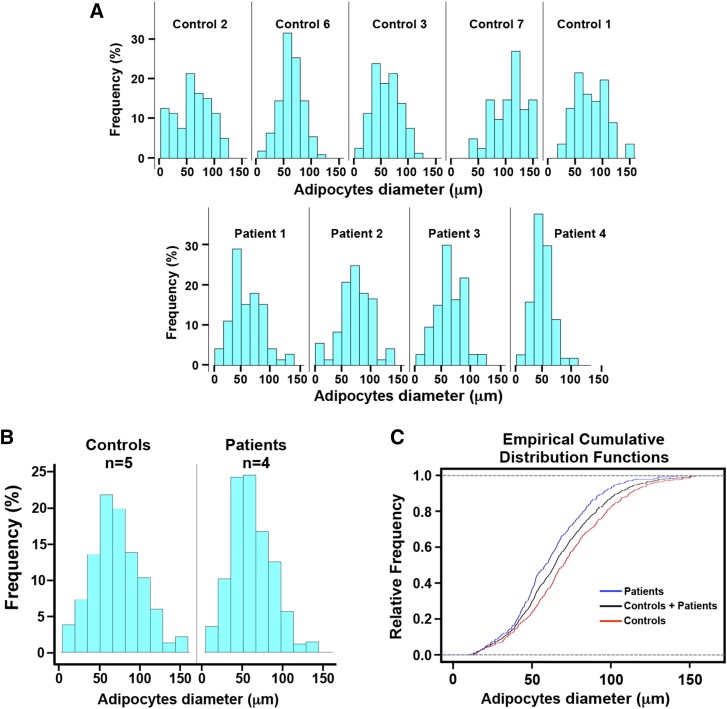
Histomorphometric analysis of adipocyte diameter in patients carrying biallelic *LPIN1* inactivating mutations. The diameter (expressed in μm) of approximately 100 adipocytes was measured in each subcutaneous adipose tissue histological section from five normal controls and four lipin-1-deficient patients. Control 1, age 12; Control 2, age 15; Control 3, age 18; Control 6, age 15; Control 7, age 15; Patient 1, age 5; Patient 2, age 5; Patient 3, age 11; Patient 4, age 47 (see Table 1). The controls were highly homogeneous in age (controls: n = 5, mean age: 15; SD = 1.9), and treated as a group. Two different statistical approaches were used to minimize the possible confounding effect of age on adipocyte size (see Results section and Tables 2, 3). A: histograms of the frequency distribution of adipocyte diameter for each control and each patient. B: histogram of the frequency distribution of adipocyte diameter for the control group (n = 5) and the patient group (n = 4). C: ECDFs of adipocytes diameter for all controls (red), all patients (blue), and the total sample (controls + patients) (black). These experiments (see Results section and Tables 2, 3) show a significant decrease in adipocyte size in patients compared with normal controls.

First, we calculated exploratory statistics for the diameter of the adipocytes for all controls and patients, suggesting a possible decrease in adipocyte diameter in patients when compared with controls (**Table 2**). Subsequently, individual histogram representations of adipocyte diameter distribution were built for each individual control and patient (**Fig. 3A**). Analogously, histograms of adipocyte diameter distribution were also built for the control and patient groups (Fig. 3B).

**TABLE 2.** Histomorphometric analysis of adipocyte diameters: exploratory statistics

Group	Minimum	1st Quartile	Median	Mean	3rd Quartile	Maximum
Controls (n = 5)	13.94	51.74	70.14	72.18	91.92	153.9
Patients (n = 4)	10.57	43.93	58.67	61.25	77.81	142.2

Exploratory statistics for the experimentally observed adipocyte diameters. The diameters (expressed in micromoles) of about 100 adipocytes were measured in each histological section derived from five normal controls and four lipin-1-defective patients, and the exploratory statistics were calculated for all five controls and for all four patients.

The empirical cumulative distribution function (ECDF) of the adipocyte diameter was then calculated for the control group versus the patient group (Fig. 3C). The adipocyte size for the patient group (n = 4) was significantly smaller than that of the normal control group (n = 5) as tested by the Kolmogorov-Smirnov nonparametric test (**Table 3** and Fig. 3C).

**TABLE 3.** Histomorphometric analysis of adipocytes diameters; Kolmogorov-Smirnov test

	D	*P*
Patients (n = 4) versus controls (n = 5)	0.174	4.59e−05*^a^*
Patient 1 versus controls (n = 5)	0.204	0.012*^b^*
Patient 2 versus controls (n = 5)	0.147	0.143
Patient 3 versus controls (n = 5)	0.157	0.092*^b^*
Patient 4 versus controls (n = 5)	0.417	1.102e−13*^a^*

The empirical cumulative distribution functions (ECDF) of the adipocytes diameters were calculated for the patient group (n = 4) and the control group (n = 5), or, to minimize the possible confounding effect of age on the adipocyte size, for each patient and for the control group, as shown in Fig. 3. The two-sample Kolmogorov-Smirnov (K-S) nonparametric test was applied to statistically test the null hypothesis that the values observed for the patient group or for each patient were drawn from the same CDF of the control group. Patient 1, age 5; Patient 2, age 5; Patient 3, age 11; patient 4, age 47. Control 1, age 12; Control 2, age 15; Control 3, age 18; Control 6, age 15; Control 7, age 15 (Controls: n = 5, mean age: 15; SD = 1.9).

a *P* < 0.001.

b *P* < 0.1.

To minimize possible confounding effects of age on adipocyte size, we adopted a special statistical analysis: the ECDFs of the adipocyte diameters were calculated for each patient and for the control group, and the Kolmogorov-Smirnov nonparametric test was used to test the null hypothesis that the values observed for each patient were drawn from the same CDF as the control group (see Table 3). Adipocyte sizes for three of four patients studied (i.e., for patient 1, age 5; for patient 3, age 11; and for patient 4, age 47) were significantly smaller than those of the normal control group (see Table 3 and Fig. 3A).

In conclusion, no significant signs of perturbation of morphology, hypoplastic development, or inflammation were observed in the adipose tissue of patients carrying *LPIN1* inactivating mutations. We found a significant (albeit moderate) reduction in adipocyte size. This latter observation indicates a slightly decreased capacity of fat accumulation in the LDs in the adipose tissue of patients harboring biallelic *LPIN1* mutations. However, we cannot exclude the possibility that the reduced adipocyte size might also depend on confounding factors, which are not fully controllable under our experimental conditions.

### Analysis of lipin expression levels and PAP activity in the adipose tissue of patients carrying biallelic *LPIN1* mutations

We also analyzed *LPIN1* gene expression levels, lipin-1 protein levels, and PAP activity in fat biopsies from human patients carrying *LPIN1* biallelic inactivating mutations. *LPIN1* deleterious mutations lead to stop codons or internal deletions [see Table 1 and (26–28, 31)]. Real-time PCR analysis showed significantly lower *LPIN1* mRNA levels in the adipose tissue from patients compared with normal controls (Fig. 6D). This could depend on an intracellular instability of the mutated/truncated *LPIN1* mRNA variants, which could be more rapidly degraded, compared with nonmutated (WT) *LPIN1* mRNA (43).

**Fig. 4.**
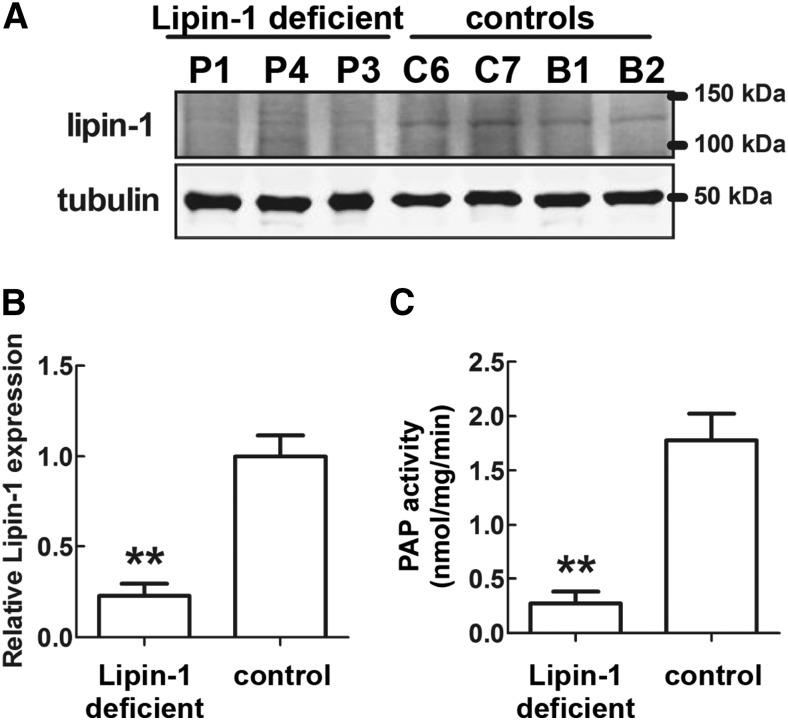
Lipin-1 protein levels and PAP activity in adipose tissue in patients carrying biallelic *LPIN1* inactivating mutations. A: Western blot analysis of lipin-1 protein levels. Sixty micrograms of total protein extracts from adipose tissue biopsies from three patients and four normal controls were subjected to Western blot analysis, with the indicated specific antibodies. Two samples of breast adipose tissue (B1 and B2) were also used as controls due to the paucity of the biopsied subcutaneous WAT controls. P1 and P4: subcutaneous WAT from Patient 1 and Patient 4 (see Table 1), carrying the *LPIN1* homozygous early stop mutations p.Arg388X/p.Arg388X; P3, subcutaneous WAT from Patient 3 (see Table 1) carrying the *LPIN1* heterzygous mutations p.Asn417LysfsX22/p.Glu766_Ser838del (i.e., an early stop mutation and a C-terminal deletion); C6 and C7, subcutaneous WAT from normal individuals (Control 6 and Control 7, see Table 1); B1 and B2: control WAT from adult breasts obtained from two normal individuals, aged 53 and 56 years, subjected to reduction mastoplasty (see Methods). B: Densitometric analysis of the relative lipin-1 protein levels equalized for tubulin expression; protein levels were obtained from Western blots in A and shown as means ± SD of three (patients) or four (controls) independent determinations. C: PAP activity for the same samples shown in A. The values in B and C for the control’s adipose tissue showed that that both measures were tightly grouped for the subcutaneous WAT controls (C6 and C7) and for the breast WAT controls (B1 and B2) and did not depend on the anatomical location of the biopsied samples. In B and C, significant differences are indicated by **P < 0.01 (Student’s *t*-test).

WAT from patients carrying *LPIN1* homozygous early stop mutations (p.Arg388X/p.Arg388X) or heterozygous *LPIN1* mutations (p.Asn417LysfsX22/p.Glu766_Ser838del, i.e., an early stop mutation and a C-terminal deletion), showed dramatically reduced levels of lipin-1 without significant differences in lipin-1 expression between homozygous and heterozygous mutant alleles (**Fig. 4A, B**). This suggests that in heterozygous patients, the mRNA resulting from the transcription of the *LPIN1* allele carrying the 3′-terminal deletion is unstable (28).

However, in none of the patients analyzed was the apparent expression of lipin-1 completely abolished: a weak protein band of about 100 kDa was still recognized in both types of *LPIN1* biallelic inactivating mutations (Fig. 4A). On average, ∼23% of the staining was still present in Western blots of WAT from patients carrying *LPIN1* mutations (Fig. 4A, B). We hypothesize that it could correspond to lipin-2 because the anti-human lipin-1 antibody that we used in the Western blot analysis can also weakly cross-react with the 98 kDa lipin-2 (results not shown).

All patients showed a similar dramatic impairment of PAP activity in their WAT with only ∼13% of the PAP activity of controls (Fig. 4C). There were no significant differences in PAP activity between the homozygous and compound heterozygous alleles. This result shows that lipin-1 accounts for the majority of PAP activity in human WAT, as expected. Previous evidence that we obtained in primary myoblasts established from lipin-1-defective patients (28) has also shown that different *LPIN1* mutant alleles (homozygous C-terminal deletion, homozygous early stop mutation, and heterozygous early stop mutation + C-terminal deletion) are all associated with a severe reduction of PAP activity, although this was not totally abolished (28).

We hypothesized that lipin-2 and lipin-3 can contribute to the residual PAP activity in lipin-1-defective patients (9, 44). Therefore, we explored the possibility of a compensatory overexpression of *LPIN2* or *LPIN3* in human adipose tissue in the presence of a deleterious mutation of *LPIN1* (9, 44). *LPIN2* mRNA expression levels were not significantly modulated in lipin-1-defective patients compared with controls (Fig. 6E). Moreover, *LPIN3* mRNA was always undetectable by real-time PCR in the adipose tissue biopsies of both patients and controls (results not shown). This is consistent with previous observations, showing that *LPIN3* is selectively expressed in intestine, platelets, B-lymphocytes, and a subset of neuronal cells, but virtually absent in other tissues like adipose and muscle [Ref. 9 and see the BioGPS gene annotation web portal (45)].

Altogether, these results suggest that: *i*) lipin-1-defective patients have severely reduced *LPIN1*/lipin-1 expression and PAP activity, *ii*) lipin-1 accounts for the majority of PAP activity in human WAT, *iii*) the expression of *LPIN2* potentially appears to provide the residual PAP activity in lipin-1-deficient patients, and *iv*) there appear to be no compensatory mechanisms that increase the expression of *LPIN2* or *LPIN3*.

### Qualitative analysis of lipids in human adipose tissue from lipin-1-defective patients

To further understand the functions of lipin-1 in human adipose tissue, we explored the qualitative composition of storage lipids in human WAT biopsies from lipin-1-defective patients.

**Fig. 5.**
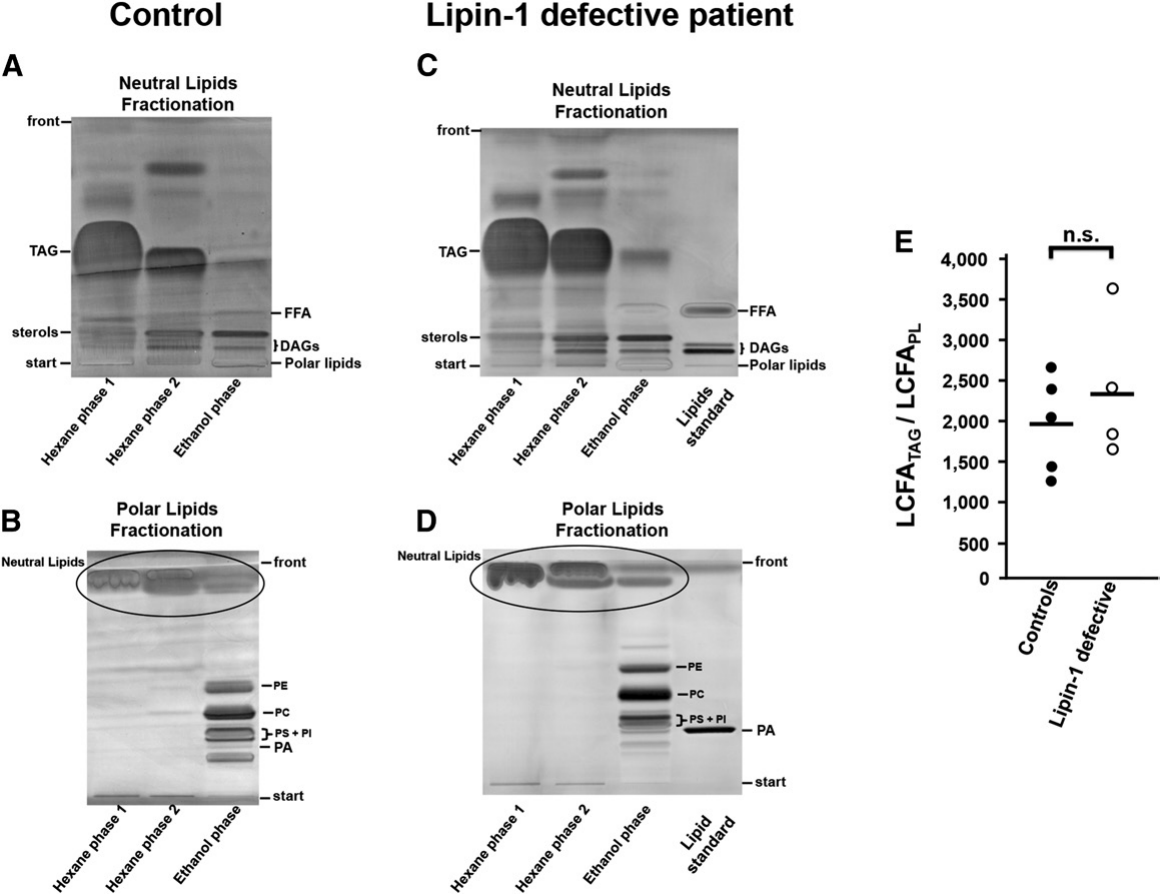
Qualitative lipid analysis of adipose tissue from lipin-1-defective patients and normal controls. A–D: representative high performance TLC of hexane and ethanol total lipid extraction fractions (34) obtained from subcutaneous WAT biopsies from a normal control individual (A, B) and from a lipin-1-defective patient (C, D). Similar experiments were repeated using adipose biopsies from five control individuals and four independent lipin-1-defective patients, obtaining analogous results. A, C: neutral lipids TLC separation. B, D: polar lipid (PL) TLC separation. DAG, diacylglycerol; FFA, unesterified (free) FAs; PA, phosphatidate; PC, phosphatidylcholine; PE, phosphatidylethanolamine; PI, phosphatidylinositol; PS, phosphatidylserine; TAG, triacylglycerol. As shown in A and C (neutral lipid TLC fractionation), the hexane extraction phases contained virtually all the TAG initially present in the total lipid extract and only part of the sterols and DAGs; the ethanol extraction phase contained the remaining part of sterols and DAGs and negligible amounts of TAG and unesterified FA. As shown in B and D (PL TLC fractionation), the hexane extraction phases were devoid of PLs and the ethanol extraction phase contained virtually all the phospholipids initially present in the total lipid extract. E: ratio LCFA_TAG_/LCFA_PL_. The relative abundance of TAG and PL in the total lipid extracts from each biopsy was determined by the internal ratio LCFA_TAG_/LCFA_PL_ corresponding to the mass (in micrograms) of LCFAs derived from TAG (LCFA_TAG_) divided by the mass (in micrograms) of LCFAs derived from whole PLs (LCFA_PL_). The silica gel zones in the TLC corresponding to TAG and PL were scraped from the respective TLC plate, and the lipids analyzed by GC-flame-ionization detection. Bars represent the means of the LCFA_TAG_/LCFA_PL_ ratio from five independent control individuals (mean = 1,959) and from four independent lipin-1 defective patients (mean = 2,355). Student’s *t*-test was used for control versus lipin-1-deficient comparison. ns, not significant.

A quantitative (i.e., absolute) determination of lipid content in the adipose tissue biopsies was difficult due to lack of tissue homogeneity among different biopsies. In fact, each minute biopsied adipose sample was characterized by a variable and uncontrollable intrinsic presence of stromal, connective, and vascular tissues, which made normalization based on the mass of protein or the total mass of each biopsy impractical. A rigorous compromise in our analysis was to quantify the relative abundance of TAG and PL in the total lipid extracts from each biopsy [**Fig. 5E** and (38)].

We applied a fully quantitative procedure to separate total PL and TAG from total lipid extracts derived from each fat biopsy (34). Representative TLCs for the extracted lipid fractions are shown for a patient and a control individual in Fig. 5A–D.

Our qualitative analysis of the lipids in human WAT showed that: *i*) inactivating mutations in the *LPIN1* gene that severely compromise the PAP activity do not significantly affect the relative proportions between TAG and PL compared with normal controls (Fig. 5E), and do not apparently affect the general qualitative lipid landscape, as shown by TLC analysis (Fig. 5A–D); *ii*) as a general physiological observation, the LCFA_TAG_ exceeds, on average, about 2,000 times the LCFA_PL_ (Fig. 5E), and the LCFA_TAG_/LCFA_PL_ ratio offers a clear estimate of the prevalence of TAG over PL in human WAT; *iii*) storage lipids from lipin-1-defective patients do not show abnormal accumulation of unesterifed FA (Fig. 5A–D); and *iv*) in TAG extracts from lipin-1-defective patients, the impairment of lipin-1 does not significantly affect the relative abundance of unsaturated and saturated long chain FAs (LCFA_TAG_ 16:0, 16:1, 18:0, 18:1, and 18:2) compared with normal control individuals (see analysis in **Table 4**).

**TABLE 4.** LCFA composition of TAGs (LCFA_TAG_) derived from lipin-1-defective patients and normal controls

LCFA_TAG_ C:D	16:0 (%)	16:1 (%)	18:0 (%)	18:1 (%)	18:2 (%)
*LPIN1* def. 1	22.82	3.82	6.37	50.03	16.96
*LPIN1* def. 3	25.30	4.91	7.15	49.19	13.45
*LPIN1* def. 4	28.20	3.10	5.90	47.10	15.50
Mean patients ± SD	25.40 ± 2.62	4.00 ± 0.84	6.46 ± 0.65	48.82 ± 1.44	15.33 ± 1.76
Control 3	33.30	4.70	4.90	46.20	10.60
Control 5	26.36	4.29	8.12	51.69	9.54
Control 6	25.71	4.92	8.41	51.53	9.43
mean controls ± SD	29.05 ± 4.28	4.86 ± 0.44	7.01 ± 1.78	48.74 ± 4.06	10.34 ± 1.20

Total lipid extracts were fractionated using a two-phase extraction method (34). TAGs were separated by TLC as shown in Fig. 3 and LCFA_TAG_ analyzed by GC. Data are presented for the different C:D (i.e., for the different lipid number, where C is the number of carbon atoms in the FA and D is the number of double bonds in the FA) as percent (%) of the total of LCFA_TAG_. Mean values ± SD for the LCFA_TAG_ (%) of patients and controls are shown for each C:D.

As mentioned before, the absence or decreased levels of PAP activity in the WAT in mouse or rat models without a functional lipin-1 results in dysregulated TAG biosynthesis, with subsequent accumulation of PA (2, 5, 22, 23). Because the very limited amount of the original WAT biopsies and the intrinsic low levels of PA, using an enzymatic method (46) or direct TLC methods (37) we could not to accurately determine the abundance of this short-lived phospholipid (Fig. 5B, D).

In conclusion, there was normal qualitative lipid composition of human WAT from lipin-1-defective patients.

### Expression analysis of key adipogenic genes in fat biopsies from lipin-1 defective patients

**Fig. 6.**
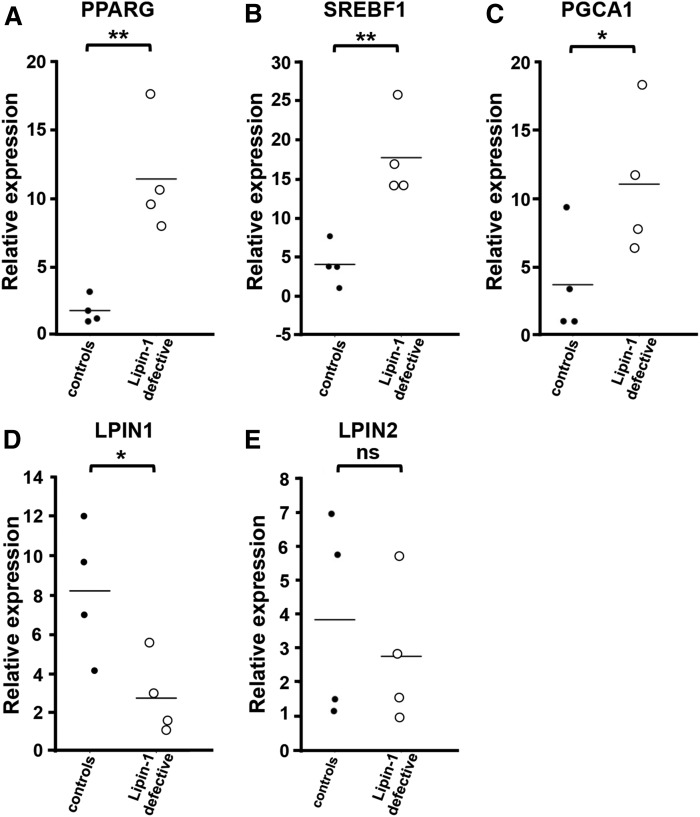
Expression analysis of key adipogenic genes in fat biopsies from lipin-1-defective patients. Subcutaneous adipose tissue biopsies from four independent lipin-1-defective patients and four independent control (healthy) individuals were collected and total RNA extracted. First-strand cDNA was generated from 1 μg total RNA to perform real-time PCR using specific primers for *PPARG* (A), *SREBF1* (B), *PGCA1* (C), *LPIN1* (D), and *LPIN2* (E). Expression data were normalized using *ACTB* as a housekeeping gene. Relative expression values are expressed as mean values (bars). ns, not significant; *P < 0.05; **P < 0.01 (Student’s *t*-test).

We performed gene-expression analysis of adipose tissue from lipin-1-defective patients with emphasis on some pivotal determinants and regulators of adipogenesis, namely *SREBF1* (SREBP1), *PPARG* (PPARγ), and *PGC1A* (PGC-1α), which have been involved in the maintenance of the mature adipose phenotype and are potential lipin-1 transcriptional targets (**Fig. 6A–C**) (47–49).

Unexpectedly, we found that fat biopsies from lipin-1-defective patients express significantly higher levels of two key transcription factors regulated by CCAAT/enhancer binding proteins (C/EBPs), namely PPARG and SREBF1, and of PGC1A, compared with normal controls (Fig. 6A–C). SREBP1 and PPARγ have an established role in FA biosynthesis and adipocyte differentiation (48), are subjected to transcriptional coactivation by PGC-1α (47, 49), and their impairment has been directly associated to congenital forms of lipodystrophy (50, 51). Therefore, our observations indicate that a specific compensatory phenomenon can be activated in vivo in human adipocytes in the presence of depleted lipin-1 expression.

### Induction of adipogenic differentiation in human dermal fibroblast populations isolated from patients carrying biallelic *LPIN1* mutations

Lipin-1 plays an established role in the regulation of mouse adipogenic differentiation (3, 7, 14, 52), and primary mouse embryonic fibroblasts derived from lipin-1-deficient mice [*Lpin1^(fld/fld)^* mice] display a severely impaired adipogenic differentiation (3).

In this study, we established primary human dermal fibroblast (HDF) populations from lipin-1-defective patients and from control individuals. It has been shown that HDF populations possess a mesenchymal potential and can be committed to adipogenic differentiation in the presence of an appropriate differentiation medium (DM) containing a combination of insulin, glucocorticoids, and pharmacological compounds that increase intracellular cAMP (53, 54). Previous investigations showed variable degrees of mesenchymal potential in HDF populations isolated from different individuals (55, 56), and this likely depends on the variable fraction of mesenchymal stem cells (MSCs) in the diverse HDF cellular preparations.

**Fig. 7.**
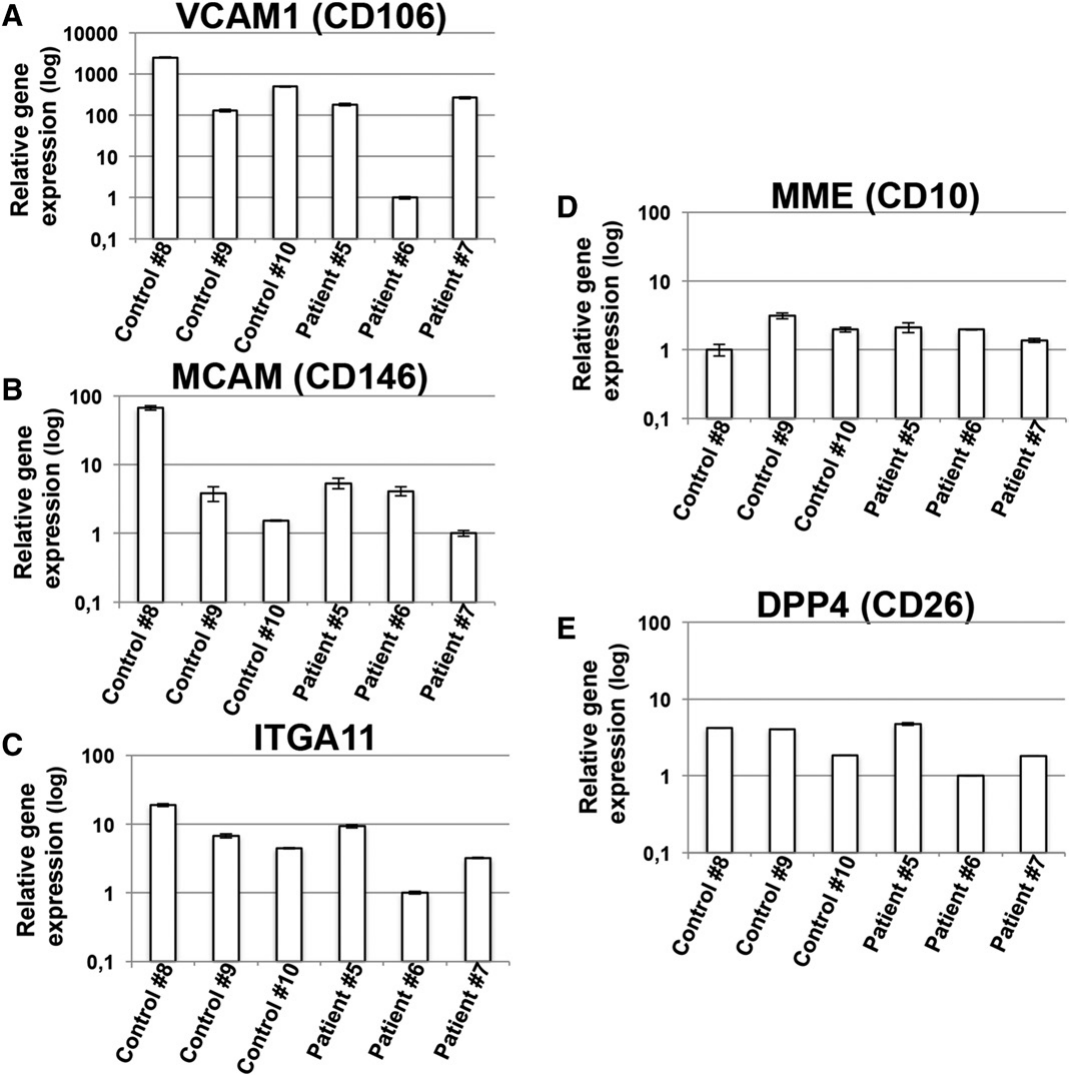
Human dermal primary fibroblast populations derived from patients carrying biallelic *LPIN1* inactivating mutations or from control individuals express MSC-specific cell surface markers. Human dermal primary fibroblast populations derived from normal control (healthy) individuals or from lipin-1-defective patients (Table 1) were cultured until confluence in GM. After 1 day of confluence, cells were harvested, total RNA extracted, and first-strand cDNA generated from 1 μg total RNA to perform real-time PCR using specific primers for *VCAM-1* (CD106) (A), *MCAM* (CD146) (B), *ITGA11* (C), *MME* (CD10) (D), and *DPP4* (CD26) (E). Expression data were normalized using *ACTB* as a housekeeping gene. Relative expression values are expressed as mean values ± SD, and shown in logarithmic scale.

Consistently, as shown in **Fig. 7**, we observed that all the different HDF populations (that we isolated from patients or controls; see Table 1) expressed significant levels of genes encoding MSC-specific surface markers [VCAM-1 (CD106), MCAM (CD146), and ITGA11 (55–57)], even when there was ample heterogeneity in the expression levels of these genes between the various HDF populations (Fig. 7). The same primary HDF populations also expressed MME (CD10) and DPP4 (CD26), which are considered to be fibroblast-specific cell surface markers (Fig. 7) (57). Therefore, all the HDF populations we isolated could potentially exhibit a mesenchymal differentiation capacity, and be potentially committed to adipogenic differentiation.

**Fig. 8.**
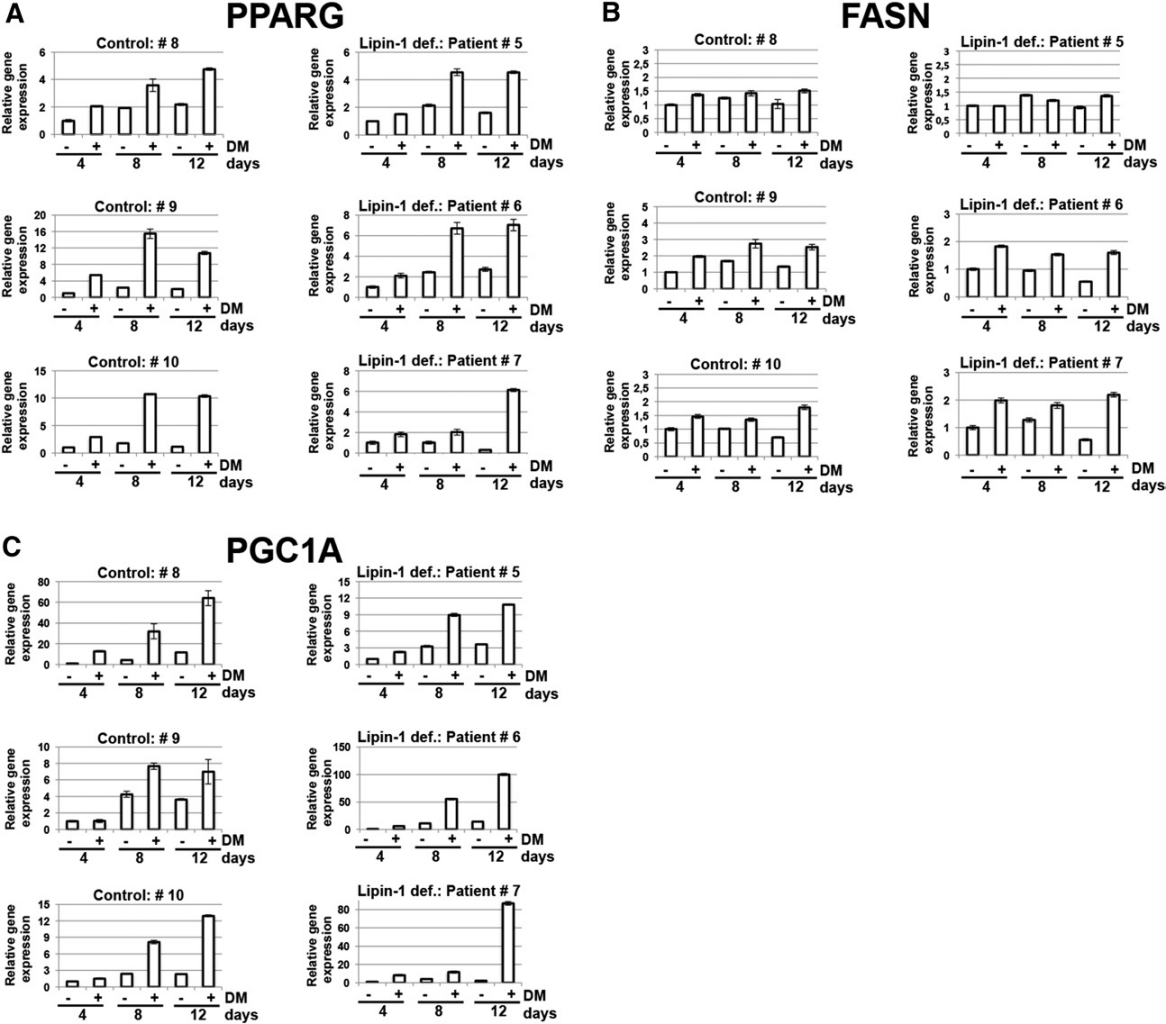
Human fibroblast populations derived from patients carrying biallelic *LPIN1* inactivating mutations can be induced to adipogenic differentiation. Human dermal primary fibroblast populations derived from normal control (healthy) individuals or from lipin-1-defective patients (see Table 1) were cultured until confluence in GM. Cells were then shifted to adipogenic differentiation medium, DM (+), containing insulin, dexamethasone, isobutylmethylxanthine, and indometacin, or left in GM (−). After 4, 8, or 12 days in DM, cells were harvested, the total RNA extracted, and first-strand cDNA generated from 1 μg of total RNA to perform real-time PCR using specific primers for *PPARG* (A), *FASN* (B), and *PGC1A* (C). Expression data were normalized using actin β (*ACTB*) as a housekeeping gene. Relative expression values are expressed as mean values ± SD.

All of the HDF populations derived from lipin-1-defective patients or normal control individuals, when exposed to adipogenic DM, progressively expressed *PPARG*, *FASN*, and *PGC1A* (**Fig. 8**), which are key markers of adipogenic differentiation (47–49, 58). This increased expression of adipogenic markers during the progression of the differentiation was associated with a late and sporadic (i.e., in less than 10% of the confluent cells) accumulation of intracellular LDs, in the late differentiation times (later than 8 days in DM; results not shown).

These results show that the impairment of lipin-1 in primary human preadipogenic cell populations does not affect the induction of key transcriptional events that characterize the multistep process of adipogenic differentiation (Fig. 8). They also show that there was a significant gene expression of the bona fide-considered MSC-specific markers (such as *VCAM-1*, *MCAM*, and *ITGA11*) in primary HDF populations, which could be a sufficient predictive indicator of adipogenic potential (56, 57). Further experiments are required to assess whether the expression of this cluster of MSC-specific markers can also outline the mesenchymal multipotency of primary HDF populations.

## DISCUSSION

We used human fat biopsies from patients carrying inactivating mutations in the *LPIN1* gene in both alleles to explore whether impairment of lipin-1 is associated with metabolic, developmental, or histopathological defects that can be ascribed to lipodystrophy or adipose tissue deficiencies. Acquired or congenital lipodystrophies are a clinically and genetically heterogeneous group of metabolic disorders characterized by either partial or generalized lack of adipose tissue (50, 51, 59). Lipodystrophies are typically associated with dyslipidemia, fatty liver, insulin resistance, diabetes, and cardiovascular disease (20, 59, 60). Some underlying genetic defects observed in lipodystrophies are directly related to adipocyte development, such as mutations in the gene encoding for the transcription factor PPARγ (50). Mutations in the gene associated with glycerolipid synthesis, such as *AGPAT2* (1-acylglycerol-3-phosphate O-acyltransferase 2, also known as *BSCL1*), are associated with congenital generalized lipodystrophy, or Berardinelli-Seip syndrome. This is a very severe form of generalized human lipodystrophy, characterized by the virtual absence of adipose tissue and a severe insulin resistance (20). *AGPAT2* encodes an enzyme located within the endoplasmic reticulum (ER) that converts lysophosphatate to phosphatate, the second step in de novo phospholipid biosynthesis. Mutations in the gene known as *BSCL2* are involved in type 2-Berardinelli-Seip congenital lipodystrophy (61). Interestingly, *BSCL2* encodes seipin, a transmembrane protein localized to the ER which can bind lipin-1 (62).

*LPIN1* is a top-rank candidate gene for human congenital lipodystrophy. Lipin-1 dephosphorylates PA to DAG, a common precursor for TAG and PL synthesis (63), acting in the metabolic pathway immediately downstream of *AGPAT2*. Therefore, the gene products of *AGPAT*, *LPIN1*, and *BSCL2* are closely functionally or physically associated, explaining how mutations in each of these genes can be directly correlated to lipodystrophy. The *Lpin1* gene is prevalently expressed in white and brown adipose tissue and skeletal and cardiac muscles, all tissues that are characterized by active TAG synthesis and/or by high capacity β-oxidation (13, 63, 64). It has been shown that adipocyte-specific *Lpin1* knockout mouse models, *Lpin1^(fld/fld)^* mice carrying spontaneous inactivation of the *Lpin1* gene, and *Lpin1^1Hubr^* rats with mutated lipin-1 protein all unambiguously display lipodystrophy (even if each animal model is characterized by different penetrance and severity of the pathological phenotype) (1, 2, 7, 14, 23, 52). The single lipin protein ortholog in *Drosophila* (encoded by *dLipin*) is also essential for normal body fat development and normal lipid accumulation and TAG storage in the LDs of adipocytes (65).

Although loss of lipin-1 in mouse models leads to manifest lipodystrophy, hepatic steatosis, and insulin resistance, so far none of these pathological conditions have also been observed in human individuals deficient in lipin-1, who have normal adipose tissue distribution and fat weight (26, 27, 31). Instead, we and others showed that deleterious *LPIN1* mutations cause recurrent severe pediatric clinical episodes of rhabdomyolysis, a syndrome resulting from the massive breakdown of skeletal muscle fibers, leading to myoglobinuria (26, 28, 31). The clinical features of rhabdomyolysis observed in human lipin-1-defective patients are compatible with those observed in *Lpin1^(fld/fld)^* mice models subjected to specific metabolic stress, in which skeletal muscle myofibrillar necrosis is also evident, due to impairment of mitochondrial functions and autophagy (29).

Until now, a thorough and systematic analysis of human adipose tissue from patients harboring *LPIN1* inactivating mutations has not been performed. We established that the WAT tissue from these patients: *i*) is characterized by severely reduced levels of *LPIN1* mRNA, lipin-1 protein and PAP activity, without the concomitant compensatory increased expression of *LPIN2* and *LPIN3;* *ii*) does not show any of the typical histopathological features associated with lipodystrophy (i.e: hypoplastic development, inflammation, increased mixoid stroma, abnormal lipid droplets, abnormal vasculature); *iii*) shows a moderate reduction of adipocyte size, indicative of a possible decreased capacity of fat accumulation in LDs; *iv*) has a normal qualitative composition of stored lipids; and *v*) expresses significantly higher levels of the key positive regulators of adipogenesis, *SREBF1*, *PPARG*, and *PGC1A*.

The observation that human adipose tissue in lipin-1-deficient patients can develop fully and store neutral lipids normally can be explained if some specific compensatory phenomena are triggered in human adipocytes. In agreement with this hypothesis, here we showed that fat biopsies from lipin-1-defective patients express significantly higher levels of *SREBP1*, *PPARG*, and *PGCA1*, three pivotal determinants of adipogenesis (63, 66). Also, mesenchymal populations derived from lipin-1-defective patients recapitulate the key transcriptional events that characterize the multistep process of adipogenic differentiation. These results agree with our previous experimental observations, showing that PPAR/PGC-1α mediated-adipogenesis is increased in human lipin-1-deficient myoblasts (28), and also with our description of an autoptic case of a young lipin-1-defective patient with an associated severe cardiac infiltration with adipocytes (67). This would be consistent with increased adipogenesis induced by *SREBP1*, *PPARG*, and *PGCA1* upregulation.

We established that lipin-1 accounts for the majority of PAP activity in human WAT, and that the inactivating mutations are compatible with normal human adipose tissue differentiation and functions. In agreement with this, depletion of lipin-1 by siRNA in the human preadipocitic SGBS cell line leads to about 95% depletion of PAP activity without impairing adipogenic differentiation and accumulation of neutral lipids (30). By contrast to what we observed in WAT of human patients carrying biallelic inactivating mutations in *LPIN1*, repression of lipin-1 in SGBS cells caused significant reduction of the key adipogenic transcription factors PPARG and SREBP1 (30).

The lipin paralogues lipin-1, -2, and -3 share homologous sequences and overlapping PAP enzymatic functions (9). Lipin-2 and -3 can potentially compensate for the absence of lipin-1 (9, 24, 68). Previous studies with 3T3-L1 mouse preadipocyte cells showed that lipin-1 is progressively expressed during differentiation in culture (6), and lipin-1 depletion after the initiation of the adipogenesis results in increased expression of lipin-2 (but not lipin-3) (25). However, in the present study, we showed that severe deficiency of lipin-1 and PAP activity in human adipose tissue was not compensated by an increased expression of *LPIN2* (Fig. 6), and *LPIN3* expression was never detectable in human adipose tissue of patients or controls (results not shown).

Virtually no PAP activity was reported in WAT and skeletal muscle from lipin-1-deficient [*Lpin1^(fld/fld)^*] mice (9). These results probably depend on the composition of the PAP assay, as adjusting the Mg^2+^ concentration and the pH in the PAP assay revealed that hearts of *Lpin1^(fld/fld)^* mice still retain about 15–20% of the PAP activity of controls (24), and this was sufficient to sustain normal rates of TAG synthesis and β-oxidation in perfused working hearts, although there was cardiac dysfunction (24). It was concluded that PAP activity is normally present in excess in mouse cardiac tissue, and that lipins-2 and -3 were able to compensate for the complete loss of lipin-1 in *Lpin1^(fld/fld)^* hearts (24).

In the present study, we used the same PAP assay used in (24) and found that WAT biopsies from lipin-1-defective patients displayed on average about 13% of the PAP activity of controls. Consistent with this, we previously showed a severe reduction of cellular PAP activity in primary myoblasts established from lipin-1-defective patients carrying different types of *LPIN1* mutant alleles (28), but we also observed that all primary myoblasts analyzed still retained significant cellular PAP activity (28). We conclude that significant residual PAP activity in WAT of lipin-1-deficient patients (Fig. 4C) depends on the low (but significant) expression of *LPIN2* in this tissue (Fig. 6). This is also substantiated by the weak residual band on the Western blots of WAT from patients, as shown in Fig. 4A and B, because the antibody anti-human lipin-1 we used cross-reacts with lipin-2 (see Methods). Using the same antibody anti-lipin-1, a residual weak protein band of about 100 kDa was also detected in Western blots of myoblasts established from the patients analyzed in this work (result not shown). The expression of lipin-2 in human adipose tissue of patients carrying lipin-1 inactivating mutations could be sufficient to sustain normal TAG synthesis and adipogenesis. This would explain the difference between the mouse and human phenotype in the absence of functional lipin-1.

Lipin-1 is a multifunctional protein that can regulate lipid metabolism at several levels (5, 10–13). Under physiological conditions and in response to increased FA delivery, lipin-1 dependent PAP activity can play a specialized role to rapidly increase the esterification of FAs into TAG in adipose tissue, whereas the lipin-1 gene regulatory effects (mediated by PGC-1α) appear to be particularly prominent in the liver, supporting the hepatic capacity for β-oxidation (FAO) and suppressing hepatic FA synthesis (13). The evidence that we have presented suggests that under homeostatic conditions, the WAT in lipin-1-defective patients shows a virtually normal metabolic lipid balance, without any evident accumulation of unesterified FAs (Fig. 5). Possibly, if these patients were challenged by circumstances of increased FA delivery (such as fasting, diabetes, a diet rich in fat, alcohol assumption), they would show the symptoms of impaired capacity for FA esterification in WAT, with a consequent accumulation of unesterified FAs.

Theoretically, LPP activity could compensate the severe PAP defect that is observed in patients. However, it was shown in an *Lpin1^1Hubr^* rat model of PAP-deficiency that the compensatory increase of LPP was restricted to the sciatic nerve endoneurium, but was not detectable in WAT (23). Also in *Lpin1^(fld/fld)^* mice, no significant difference in LPP activity was found in WAT (22). Furthermore, the location of the catalytic site of the LPPs on the outer surface of the plasma membrane or on the luminal surface of internal membranes make it unlikely that the PA formed during lipid synthesis in the ER could be available directly to the LPPs (69).

Lipin-1 catalyzes a crucial step in TAG biosynthesis, which agrees with the reduced neutral lipid storage and accumulation of PA that has been observed in lipin-1-deficient mice (2, 5, 7, 14, 21–23). Analysis of the lipids in the muscle of *Lpin1^(fld/fld)^* mice showed accumulation of high levels of various phospholipids and unesterified FA, that could contribute to altered metabolism in lipin-1-deficient muscles (29). Our analysis of WAT biopsies revealed that patients carrying inactivating mutations of the *LPIN1* gene are not characterized by altered relative proportions between TAG and PL, or by altered relative proportion of the long chain FA in the TAG, or by abnormal accumulation of unesterified FA or PLs (Fig. 5).

We found a significant but moderate reduction in adipocyte size in lipin-1-deficient patients compared with normal controls, which could be symptomatic of reduced capacity for neutral lipid storage in the LDs in these patients. However, we cannot completely exclude the possibility that reduced adipocyte size might depend on other confounding factors (age, genetic background, environmental factors, etc.) or adaptations of energy balance linked to muscle alteration in patients. Accumulation of neutral lipids is determined by balance among lipid biosynthesis, lipid hydrolysis, and β-oxidation. Therefore, further analysis is required to explore the hypothesis that other nonlipin-dependent compensatory pathways, like the monoacylglycerol O-acyltransferase (MOGAT) pathway (70), or the downregulation of the lipid hydrolysis and FAO, may contribute to compensate for the deficiency of lipin-1 in human patients.

In summary, adipose tissue from patients with biallelic *LPIN1* inactivating mutations displays a dramatic reduction in lipin-1 levels and PAP activity. Nevertheless, adipose tissue appeared to develop normally without manifest signs of lipodystrophy and with a normal qualitative composition of storage lipids. The overexpression of key adipogenic determinants such as *SREBP1*, *PPARG*, and *PGC1A* and residual PAP activity suggest that specific compensatory phenomena should be activated in human adipocytes in the presence of lipin-1 depletion. Additional work is required to decipher the exact nature of this compensation circumstance. This paper highlights the evidence that human beings and mice can exhibit very important patho-physiological differences, despite very high conservation of genomes and great similarity in transcriptional landscapes, gene regulation machinery, and metabolic networks. In particular, the penetrance of deleterious mutations in metabolic genes can depend greatly on the activation of compensatory phenomena such as metabolic and genetic regulations, expression of paralogues genes, etc., in association with environmental factors.

## References

[1] Langner C. A., Birkenmeier E. H., Ben-Zeev O., Schotz M. C., Sweet H. O., Davisson M. T., and Gordon J. I. 1989 The fatty liver dystrophy (fld) mutation. A new mutant mouse with a developmental abnormality in triglyceride metabolism and associated tissue-specific defects in lipoprotein lipase and hepatic lipase activities. J. Biol. Chem. 264: 7994–8003. 2722772

[2] Reue K. 2007 The role of lipin 1 in adipogenesis and lipid metabolism. Novartis Found. Symp. 286: 58–68; discussion 68–71, 162–163, 196–203. 18269174 10.1002/9780470985571.ch6

[3] Phan J., Peterfy M., and Reue K. 2004 Lipin expression preceding peroxisome proliferator-activated receptor-gamma is critical for adipogenesis in vivo and in vitro. J. Biol. Chem. 279: 29558–29564. 15123608 10.1074/jbc.M403506200

[4] Phan J., Peterfy M., and Reue K. 2005 Biphasic expression of lipin suggests dual roles in adipocyte development. Drug News Perspect. 18: 5–11. 15753971 10.1358/dnp.2005.18.1.877165

[5] Mitra M. S., Chen Z., Ren H., Harris T. E., Chambers K. T., Hall A. M., Nadra K., Klein S., Chrast R., Su X., 2013 Mice with an adipocyte-specific lipin 1 separation-of-function allele reveal unexpected roles for phosphatidic acid in metabolic regulation. Proc. Natl. Acad. Sci. USA. 110: 642–647. 23267081 10.1073/pnas.1213493110 PMC3545773

[6] Péeterfy M., Phan J., and Reue K. 2005 Alternatively spliced lipin isoforms exhibit distinct expression pattern, subcellular localization, and role in adipogenesis. J. Biol. Chem. 280: 32883–32889. 16049017 10.1074/jbc.M503885200

[7] Péterfy M., Phan J., Xu P., and Reue K. 2001 Lipodystrophy in the fld mouse results from mutation of a new gene encoding a nuclear protein, lipin. Nat. Genet. 27: 121–124. 11138012 10.1038/83685

[8] Han G. S., Wu W. I., and Carman G. M. 2006 The Saccharomyces cerevisiae lipin homolog is a Mg2+-dependent phosphatidate phosphatase enzyme. J. Biol. Chem. 281: 9210–9218. 16467296 10.1074/jbc.M600425200 PMC1424669

[9] Donkor J., Sariahmetoglu M., Dewald J., Brindley D. N., and Reue K. 2007 Three mammalian lipins act as phosphatidate phosphatases with distinct tissue expression patterns. J. Biol. Chem. 282: 3450–3457. 17158099 10.1074/jbc.M610745200

[10] Pascual F., and Carman G. M. 2013 Phosphatidate phosphatase, a key regulator of lipid homeostasis. Biochim. Biophys. Acta. 1831: 514–522. 22910056 10.1016/j.bbalip.2012.08.006 PMC3549317

[11] Sugden M. C., Caton P. W., and Holness M. J. 2010 PPAR control: it’s SIRTainly as easy as PGC. J. Endocrinol. 204: 93–104. 19770177 10.1677/JOE-09-0359

[12] Peterson T. R., Sengupta S. S., Harris T. E., Carmack A. E., Kang S. A., Balderas E., Guertin D. A., Madden K. L., Carpenter A. E., Finck B. N., 2011 mTOR complex 1 regulates lipin 1 localization to control the SREBP pathway. Cell. 146: 408–420. 21816276 10.1016/j.cell.2011.06.034 PMC3336367

[13] Finck B. N., Gropler M. C., Chen Z., Leone T. C., Croce M. A., Harris T. E., Lawrence J. C. Jr., and Kelly D. P. 2006 Lipin 1 is an inducible amplifier of the hepatic PGC-1alpha/PPARalpha regulatory pathway. Cell Metab. 4: 199–210. 16950137 10.1016/j.cmet.2006.08.005

[14] Nadra K., Medard J. J., Mul J. D., Han G. S., Gres S., Pende M., Metzger D., Chambon P., Cuppen E., Saulnier-Blache J. S., 2012 Cell autonomous lipin 1 function is essential for development and maintenance of white and brown adipose tissue. Mol. Cell. Biol. 32: 4794–4810. 23028044 10.1128/MCB.00512-12 PMC3497595

[15] Kim H. B., Kumar A., Wang L., Liu G. H., Keller S. R., Lawrence J. C. Jr., Finck B. N., and Harris T. E. 2010 Lipin 1 represses NFATc4 transcriptional activity in adipocytes to inhibit secretion of inflammatory factors. Mol. Cell. Biol. 30: 3126–3139. 20385772 10.1128/MCB.01671-09 PMC2876672

[16] Rehnmark S., Giometti C. S., Slavin B. G., Doolittle M. H., and Reue K. 1998 The fatty liver dystrophy mutant mouse: microvesicular steatosis associated with altered expression levels of peroxisome proliferator-regulated proteins. J. Lipid Res. 39: 2209–2217. 9799807

[17] Reue K., and Zhang P. 2008 The lipin protein family: dual roles in lipid biosynthesis and gene expression. FEBS Lett. 582: 90–96. 18023282 10.1016/j.febslet.2007.11.014 PMC2848953

[18] Kanda H., Tateya S., Tamori Y., Kotani K., Hiasa K., Kitazawa R., Kitazawa S., Miyachi H., Maeda S., Egashira K., 2006 MCP-1 contributes to macrophage infiltration into adipose tissue, insulin resistance, and hepatic steatosis in obesity. J. Clin. Invest. 116: 1494–1505. 16691291 10.1172/JCI26498 PMC1459069

[19] Meana C., Pena L., Lorden G., Esquinas E., Guijas C., Valdearcos M., Balsinde J., and Balboa M. A. 2014 Lipin-1 integrates lipid synthesis with proinflammatory responses during TLR activation in macrophages. J. Immunol. 193: 4614–4622. 25252959 10.4049/jimmunol.1400238

[20] Agarwal A. K., Arioglu E., De Almeida S., Akkoc N., Taylor S. I., Bowcock A. M., Barnes R. I., and Garg A. 2002 AGPAT2 is mutated in congenital generalized lipodystrophy linked to chromosome 9q34. Nat. Genet. 31: 21–23. 11967537 10.1038/ng880

[21] Takeuchi K., and Reue K. 2009 Biochemistry, physiology, and genetics of GPAT, AGPAT, and lipin enzymes in triglyceride synthesis. Am. J. Physiol. Endocrinol. Metab. 296: E1195–E1209. 19336658 10.1152/ajpendo.90958.2008 PMC2692402

[22] Nadra K., de Preux Charles A. S., Medard J. J., Hendriks W. T., Han G. S., Gres S., Carman G. M., Saulnier-Blache J. S., Verheijen M. H., and Chrast R. 2008 Phosphatidic acid mediates demyelination in Lpin1 mutant mice. Genes Dev. 22: 1647–1661. 18559480 10.1101/gad.1638008 PMC2428062

[23] Mul J. D., Nadra K., Jagalur N. B., Nijman I. J., Toonen P. W., Medard J. J., Gres S., de Bruin A., Han G. S., Brouwers J. F., 2011 A hypomorphic mutation in lpin1 induces progressively improving neuropathy and lipodystrophy in the rat. J. Biol. Chem. 286: 26781–26793. 21715287 10.1074/jbc.M110.197947 PMC3143639

[24] Kok B. P., Kienesberger P. C., Dyck J. R., and Brindley D. N. 2012 Relationship of glucose and oleate metabolism to cardiac function in lipin-1 deficient (fld) mice. J. Lipid Res. 53: 105–118. 22058427 10.1194/jlr.M019430 PMC3243467

[25] Sembongi H., Miranda M., Han G. S., Fakas S., Grimsey N., Vendrell J., Carman G. M., and Siniossoglou S. 2013 Distinct roles of the phosphatidate phosphatases lipin 1 and 2 during adipogenesis and lipid droplet biogenesis in 3T3–L1 cells. J. Biol. Chem. 288: 34502–34513. 24133206 10.1074/jbc.M113.488445 PMC3843065

[26] Zeharia A., Shaag A., Houtkooper R. H., Hindi T., de Lonlay P., Erez G., Hubert L., Saada A., de Keyzer Y., Eshel G., 2008 Mutations in LPIN1 cause recurrent acute myoglobinuria in childhood. Am. J. Hum. Genet. 83: 489–494. 18817903 10.1016/j.ajhg.2008.09.002 PMC2561931

[27] Michot C., Hubert L., Brivet M., De Meirleir L., Valayannopoulos V., Muller-Felber W., Venkateswaran R., Ogier H., Desguerre I., Altuzarra C., 2010 LPIN1 gene mutations: a major cause of severe rhabdomyolysis in early childhood. Hum. Mutat. 31: E1564–E1573. 20583302 10.1002/humu.21282

[28] Michot C., Mamoune A., Vamecq J., Viou M. T., Hsieh L. S., Testet E., Laine J., Hubert L., Dessein A. F., Fontaine M., 2013 Combination of lipid metabolism alterations and their sensitivity to inflammatory cytokines in human lipin-1-deficient myoblasts. Biochim. Biophys. Acta. 1832: 2103–2114. 23928362 10.1016/j.bbadis.2013.07.021 PMC4007099

[29] Zhang P., Verity M. A., and Reue K. 2014 Lipin-1 regulates autophagy clearance and intersects with statin drug effects in skeletal muscle. Cell Metab. 20: 267–279. 24930972 10.1016/j.cmet.2014.05.003 PMC4170588

[30] Temprano A., Sembongi H., Han G. S., Sebastian D., Capellades J., Moreno C., Guardiola J., Wabitsch M., Richart C., Yanes O., 2016 Redundant roles of the phosphatidate phosphatase family in triacylglycerol synthesis in human adipocytes. Diabetologia. 59: 1985–1994. 27344312 10.1007/s00125-016-4018-0 PMC4969345

[31] Michot C., Hubert L., Romero N. B., Gouda A., Mamoune A., Mathew S., Kirk E., Viollet L., Rahman S., Bekri S., 2012 Study of LPIN1, LPIN2 and LPIN3 in rhabdomyolysis and exercise-induced myalgia. J. Inherit. Metab. Dis. 35: 1119–1128. 22481384 10.1007/s10545-012-9461-6

[32] Briand N., Le Lay S., Sessa W. C., Ferre P., and Dugail I. 2011 Distinct roles of endothelial and adipocyte caveolin-1 in macrophage infiltration and adipose tissue metabolic activity. Diabetes. 60: 448–453. 21270257 10.2337/db10-0856 PMC3028344

[33] Pelosi M., Alfò M., Martella F., Pappalardo E., and Musarò A. 2015 Finite mixture clustering of human tissues with different levels of IGF-1 splice variants mRNA transcripts. BMC Bioinformatics. 16: 289. 26370240 10.1186/s12859-015-0689-7 PMC4570607

[34] Galanos D. S., and Kapoulas V. M. 1965 Preparation and analysis of lipid extracts from milk and other tissues. Biochim. Biophys. Acta. 98: 278–292. 14320222 10.1016/0005-2760(65)90121-9

[35] Juguelin H., Heape A., Boiron F., and Cassagne C. 1986 A quantitative developmental study of neutral lipids during myelinogenesis in the peripheral nervous system of normal and trembler mice. Brain Res. 390: 249–252. 3955373 10.1016/s0006-8993(86)80233-5

[36] Vitiello F., and Zanetta J. P. 1978 Thin-layer chromatography of phospholipids. J. Chromatogr . 166: 637–640. 748365 10.1016/s0021-9673(00)95654-1

[37] Handloser D., Widmer V., and Reich E. 2008 Separation of phospholipids by HPTLC – an investigation of important parameters. J. Liq. Chromatogr. Relat. Technol. 31: 1857–1870.

[38] Testet E., Laroche-Traineau J., Noubhani A., Coulon D., Bunoust O., Camougrand N., Manon S., Lessire R., and Bessoule J. J. 2005 Ypr140wp, ‘the yeast tafazzin’, displays a mitochondrial lysophosphatidylcholine (lyso-PC) acyltransferase activity related to triacylglycerol and mitochondrial lipid synthesis. Biochem. J. 387: 617–626. 15588229 10.1042/BJ20041491 PMC1134991

[39] Mamoune A., Bahuau M., Hamel Y., Serre V., Pelosi M., Habarou F., Nguyen Morel M. A., Boisson B., Vergnaud S., Viou M. T., 2014 A thermolabile aldolase A mutant causes fever-induced recurrent rhabdomyolysis without hemolytic anemia. PLoS Genet. 10: e1004711. 25392908 10.1371/journal.pgen.1004711 PMC4230727

[40] Pelosi M., De Rossi M., Barberi L., and Musarò A. 2014 IL-6 impairs myogenic differentiation by downmodulation of p90RSK/eEF2 and mTOR/p70S6K axes, without affecting AKT activity. BioMed Res. Int. 2014: 206026. 24967341 10.1155/2014/206026 PMC4055274

[41] Winkelmann R. K., and Frigas E. 1986 Eosinophilic panniculitis: a clinicopathologic study. J. Cutan. Pathol. 13: 1–12. 3700770 10.1111/j.1600-0560.1986.tb00455.x

[42] Greenberg A. S., Egan J. J., Wek S. A., Garty N. B., Blanchette-Mackie E. J., and Londos C. 1991 Perilipin, a major hormonally regulated adipocyte-specific phosphoprotein associated with the periphery of lipid storage droplets. J. Biol. Chem. 266: 11341–11346. 2040638

[43] Hentze M. W., and Kulozik A. E. 1999 A perfect message: RNA surveillance and nonsense-mediated decay. Cell. 96: 307–310. 10025395 10.1016/s0092-8674(00)80542-5

[44] Dwyer J. R., Donkor J., Zhang P., Csaki L. S., Vergnes L., Lee J. M., Dewald J., Brindley D. N., Atti E., Tetradis S., 2012 Mouse lipin-1 and lipin-2 cooperate to maintain glycerolipid homeostasis in liver and aging cerebellum. Proc. Natl. Acad. Sci. USA. 109: E2486–E2495. 22908270 10.1073/pnas.1205221109 PMC3443145

[45] Wu C., Orozco C., Boyer J., Leglise M., Goodale J., Batalov S., Hodge C. L., Haase J., Janes J., Huss J. W. III, 2009 BioGPS: an extensible and customizable portal for querying and organizing gene annotation resources. Genome Biol. 10: R130. 19919682 10.1186/gb-2009-10-11-r130 PMC3091323

[46] Morita S. Y., Ueda K., and Kitagawa S. 2009 Enzymatic measurement of phosphatidic acid in cultured cells. J. Lipid Res. 50: 1945–1952. 19369695 10.1194/jlr.D900014-JLR200 PMC2724771

[47] Rosen E. D., Hsu C. H., Wang X., Sakai S., Freeman M. W., Gonzalez F. J., and Spiegelman B. M. 2002 C/EBPalpha induces adipogenesis through PPARgamma: a unified pathway. Genes Dev. 16: 22–26. 11782441 10.1101/gad.948702 PMC155311

[48] Kim J. B., Sarraf P., Wright M., Yao K. M., Mueller E., Solanes G., Lowell B. B., and Spiegelman B. M. 1998 Nutritional and insulin regulation of fatty acid synthetase and leptin gene expression through ADD1/SREBP1. J. Clin. Invest. 101: 1–9. 9421459 10.1172/JCI1411 PMC508533

[49] Summermatter S., Baum O., Santos G., Hoppeler H., and Handschin C. 2010 Peroxisome proliferator-activated receptor {gamma} coactivator 1{alpha} (PGC-1{alpha}) promotes skeletal muscle lipid refueling in vivo by activating de novo lipogenesis and the pentose phosphate pathway. J. Biol. Chem. 285: 32793–32800. 20716531 10.1074/jbc.M110.145995 PMC2963391

[50] Hegele R. A., Cao H., Frankowski C., Mathews S. T., and Leff T. 2002 PPARG F388L, a transactivation-deficient mutant, in familial partial lipodystrophy. Diabetes. 51: 3586–3590. 12453919 10.2337/diabetes.51.12.3586

[51] Agarwal A. K., and Garg A. 2002 A novel heterozygous mutation in peroxisome proliferator-activated receptor-gamma gene in a patient with familial partial lipodystrophy. J. Clin. Endocrinol. Metab. 87: 408–411. 11788685 10.1210/jcem.87.1.8290

[52] Reue K., Xu P., Wang X. P., and Slavin B. G. 2000 Adipose tissue deficiency, glucose intolerance, and increased atherosclerosis result from mutation in the mouse fatty liver dystrophy (fld) gene. J. Lipid Res. 41: 1067–1076. 10884287

[53] Russell T. R., and Ho R. 1976 Conversion of 3T3 fibroblasts into adipose cells: triggering of differentiation by prostaglandin F2alpha and 1-methyl-3-isobutyl xanthine. Proc. Natl. Acad. Sci. USA. 73: 4516–4520. 188043 10.1073/pnas.73.12.4516 PMC431523

[54] Haniffa M. A., Wang X. N., Holtick U., Rae M., Isaacs J. D., Dickinson A. M., Hilkens C. M., and Collin M. P. 2007 Adult human fibroblasts are potent immunoregulatory cells and functionally equivalent to mesenchymal stem cells. J. Immunol. 179: 1595–1604. 17641026 10.4049/jimmunol.179.3.1595

[55] Brendel C., Kuklick L., Hartmann O., Kim T. D., Boudriot U., Schwell D., and Neubauer A. 2005 Distinct gene expression profile of human mesenchymal stem cells in comparison to skin fibroblasts employing cDNA microarray analysis of 9600 genes. Gene Expr. 12: 245–257. 16355723 10.3727/000000005783992043 PMC6009126

[56] Halfon S., Abramov N., Grinblat B., and Ginis I. 2011 Markers distinguishing mesenchymal stem cells from fibroblasts are downregulated with passaging. Stem Cells Dev. 20: 53–66. 20528146 10.1089/scd.2010.0040

[57] Cappellesso-Fleury S., Puissant-Lubrano B., Apoil P. A., Titeux M., Winterton P., Casteilla L., Bourin P., and Blancher A. 2010 Human fibroblasts share immunosuppressive properties with bone marrow mesenchymal stem cells. J. Clin. Immunol. 30: 607–619. 20405178 10.1007/s10875-010-9415-4

[58] Kim J. B., and Spiegelman B. M. 1996 ADD1/SREBP1 promotes adipocyte differentiation and gene expression linked to fatty acid metabolism. Genes Dev. 10: 1096–1107. 8654925 10.1101/gad.10.9.1096

[59] Agarwal A. K., Simha V., Oral E. A., Moran S. A., Gorden P., O’Rahilly S., Zaidi Z., Gurakan F., Arslanian S. A., Klar A., 2003 Phenotypic and genetic heterogeneity in congenital generalized lipodystrophy. J. Clin. Endocrinol. Metab. 88: 4840–4847. 14557463 10.1210/jc.2003-030855

[60] Yao-Borengasser A., Rasouli N., Varma V., Miles L. M., Phanavanh B., Starks T. N., Phan J., Spencer H. J. III, McGehee R. E. Jr., Reue K., 2006 Lipin expression is attenuated in adipose tissue of insulin-resistant human subjects and increases with peroxisome proliferator-activated receptor gamma activation. Diabetes. 55: 2811–2818. 17003347 10.2337/db05-1688

[61] Magré J., Delépine M., Khallouf E., Gedde-Dahl T. Jr., Van Maldergem L., Sobel E., Papp J., Meier M., Mégarbané A., Bachy A., 2001 Identification of the gene altered in Berardinelli-Seip congenital lipodystrophy on chromosome 11q13. Nat. Genet. 28: 365–370. 11479539 10.1038/ng585

[62] Sim M. F., Dennis R. J., Aubry E. M., Ramanathan N., Sembongi H., Saudek V., Ito D., O’Rahilly S., Siniossoglou S., and Rochford J. J. 2012 The human lipodystrophy protein seipin is an ER membrane adaptor for the adipogenic PA phosphatase lipin 1. Mol. Metab. 2: 38–46. 24024128 10.1016/j.molmet.2012.11.002 PMC3757660

[63] Donkor J., Sparks L. M., Xie H., Smith S. R., and Reue K. 2008 Adipose tissue lipin-1 expression is correlated with peroxisome proliferator-activated receptor alpha gene expression and insulin sensitivity in healthy young men. J. Clin. Endocrinol. Metab. 93: 233–239. 17925338 10.1210/jc.2007-1535 PMC2190746

[64] Mitra M. S., Schilling J. D., Wang X., Jay P. Y., Huss J. M., Su X., and Finck B. N. 2011 Cardiac lipin 1 expression is regulated by the peroxisome proliferator activated receptor gamma coactivator 1alpha/estrogen related receptor axis. J. Mol. Cell. Cardiol. 51: 120–128. 21549711 10.1016/j.yjmcc.2011.04.009 PMC3104300

[65] Ugrankar R., Liu Y., Provaznik J., Schmitt S., and Lehmann M. 2011 Lipin is a central regulator of adipose tissue development and function in Drosophila melanogaster. Mol. Cell. Biol. 31: 1646–1656. 21300783 10.1128/MCB.01335-10 PMC3126333

[66] Zhang P., Takeuchi K., Csaki L. S., and Reue K. 2012 Lipin-1 phosphatidic phosphatase activity modulates phosphatidate levels to promote peroxisome proliferator-activated receptor gamma (PPARgamma) gene expression during adipogenesis. J. Biol. Chem. 287: 3485–3494. 22157014 10.1074/jbc.M111.296681 PMC3271002

[67] Bergounioux J., Brassier A., Rambaud C., Bustarret O., Michot C., Hubert L., Arnoux J. B., Laquerriere A., Bekri S., Galene-Gromez S., 2012 Fatal rhabdomyolysis in 2 children with LPIN1 mutations. J. Pediatr. 160: 1052–1054. 22480698 10.1016/j.jpeds.2012.02.033

[68] Zhou J., and Young T. L. 2005 Evaluation of Lipin 2 as a candidate gene for autosomal dominant 1 high-grade myopia. Gene. 352: 10–19. 15862761 10.1016/j.gene.2005.02.019

[69] Kok B. P. C., Venkatraman G., Capatos D., and Brindley D. N. 2012 Unlike two peas in a pod: lipid phosphate phosphatases and phosphatidate phosphatases. Chem. Rev. 112: 5121–5146. 22742522 10.1021/cr200433m

[70] Shi Y., and Cheng D. 2009 Beyond triglyceride synthesis: the dynamic functional roles of MGAT and DGAT enzymes in energy metabolism. Am. J. Physiol. Endocrinol. Metab. 297: E10–E18. 19116371 10.1152/ajpendo.90949.2008 PMC3735925

